# Supplementary material for: An autonomous organic reaction search engine for chemical reactivity
Source: Nat Commun. 2017 Jun 9;8:15733. doi: 10.1038/ncomms15733 (PMC5472751; doi:10.1038/ncomms15733)
Supplement: Supplementary Information — Supplementary figures, supplementary tables and supplementary notes. [file ncomms15733-s1.pdf]

Supplementary Tables

Supplementary Table 1. Specifications of the bench-top mass spectrometer.

Mass analyzer	ionchip® quadrupole mass spectrometer
Direct flow rate	0.2 μL min^{-1}– 2 μL min^{-1}
Split flow rate	up to 2.0 mL min^{-1}
Make-up flow	1 mL min^{-1}, 50 : 50 MeOH : H_2O
Attenuation	1000
Ionisation mode	positive
Tip voltage	850 V
Nebulizer (N_2) flow	2.5 L min^{-1}
Vacuum interface voltage	40 V
Tube lens voltage	10 V
Plate lens voltage	5 V
Ion guide voltage	1 V
Count time	0.20 ms
Mass range	*m/z* 50-800 with ionchip®150
Mass accuracy	+/- *m/z* 0.3 in full scan
Mass resolution	*m/z* 0.7 +/- 0.1 FWHM

Supplementary Table 2. Summary of the setup characteristics.

**Number of pumps**	14
**Volume of the syringes**	5 mL
**Number of reactors**	3 (R1, R2 and R2)
**Reactor volume**	R1 and R2: 1.5 mL<br>R3: 3.0 mL
**Reactor type**	R1 and R2: 1/16” OD PTFE tubing with an internal diameter of 1.0 mm and a tube length of 1.91 m.<br>R3: 1/16” OD PTFE tubing with an internal diameter of 1.0 mm and a tube length of 3.82 m.
**Connectors**	Standard connectors made of FPM and PEEK equipped with check valves (made of PEEK with a Chemraz® O-ring, which is compatible with organic solvents and compounds)
**In-line analysis**	ATR-IR and ESI-MS
**VI control**	Pathway Dependent Chemistry

Supplementary Table 3. Calibration curve of the starting material **1**, calculated processing IR spectra of this compound as a THF solution at different concentrations.

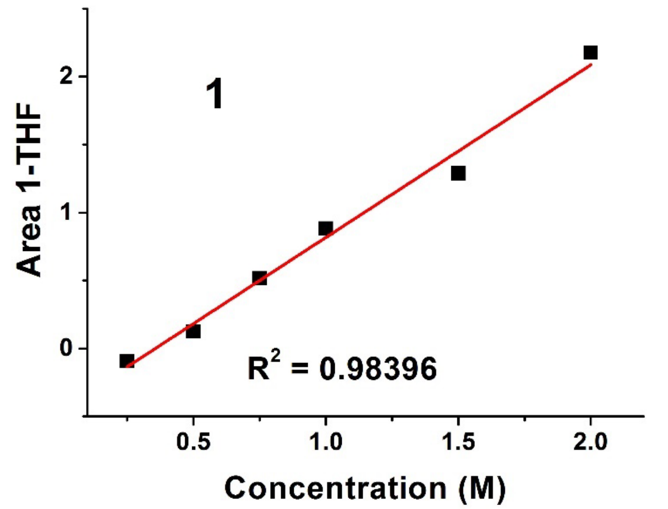

Concentration / M	Area*
2	2.17717
1.5	1.28914
1	0.88415
0.75	0.51827
0.50	0.12637
0.25	-0.09187

Supplementary Table 4. Calibration curve of the starting material **2a**, calculated processing IR spectra of this compound as a THF solution at different concentrations.

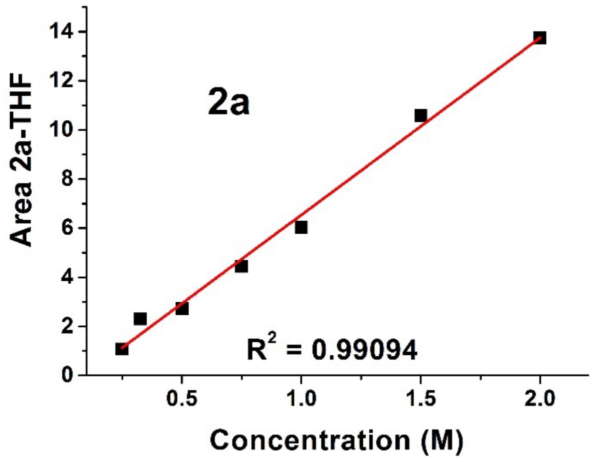

Concentration / M	Area*
2	13.73787
1.5	10.58803
1	6.03767
0.75	4.44602
0.50	2.71861
0.325	2.31428
0.25	1.07541

Supplementary Table 5. Calibration curve of the starting material **2b**, calculated processing IR spectra of this compound as a THF solution at different concentrations.

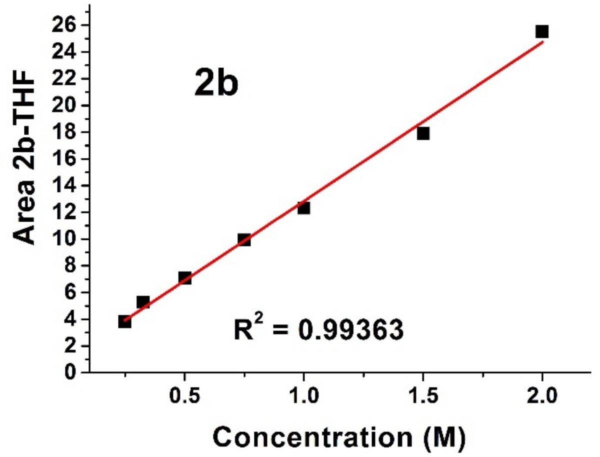

Concentration / M	Area*
2	25.51417
1.5	17.92191
1	12.3231
0.75	9.91491
0.50	7.08733
0.325	5.28701
0.25	3.84204

Supplementary Table 6. Calibration curve of the starting material **2c**, calculated processing IR spectra of this compound as a THF solution at different concentrations.

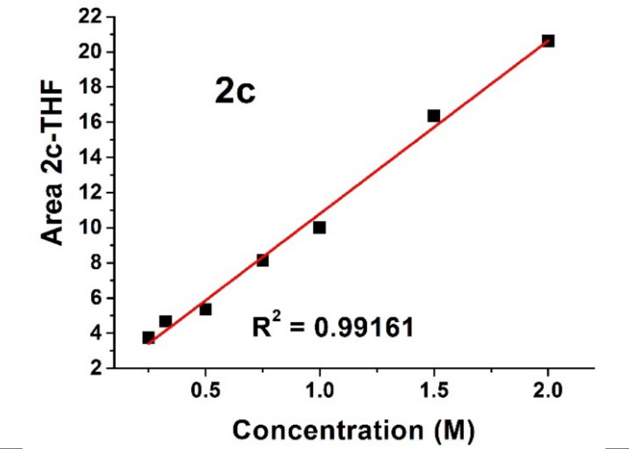

Concentration / M	Area*
2	20.61569
1.5	16.35857
1	10.01078
0.75	8.14723
0.50	5.35569
0.325	4.67593
0.25	3.74456

Supplementary Table 7. Calibration curve of the starting material **2d**, calculated processing IR spectra of this compound as a THF solution at different concentrations.

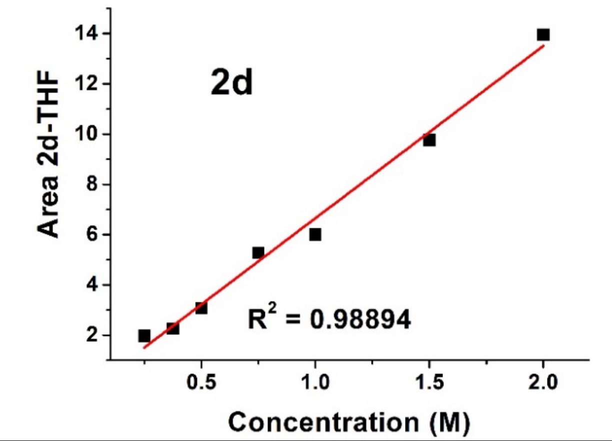

Concentration / M	Area*
2	13.94552
1.5	9.76181
1	6.00236
0.75	5.27559
0.50	3.07334
0.325	2.2585
0.25	1.96953

Supplementary Table 8. Calibration curve of the starting material **4a**, calculated processing IR spectra of this compound as a THF solution at different concentrations.

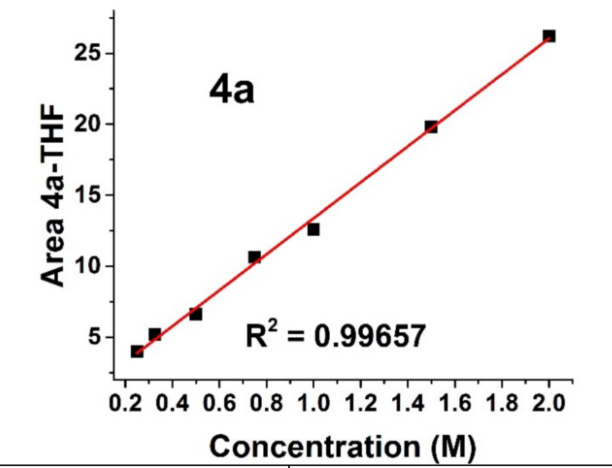

Concentration / M	Area*
2	26.21908
1.5	19.80857
1	12.59837
0.75	10.6449
0.50	6.62125
0.325	5.19117
0.25	3.98109

Supplementary Table 9. Calibration curve of the starting material **4b**, calculated processing IR spectra of this compound as a THF solution at different concentrations.

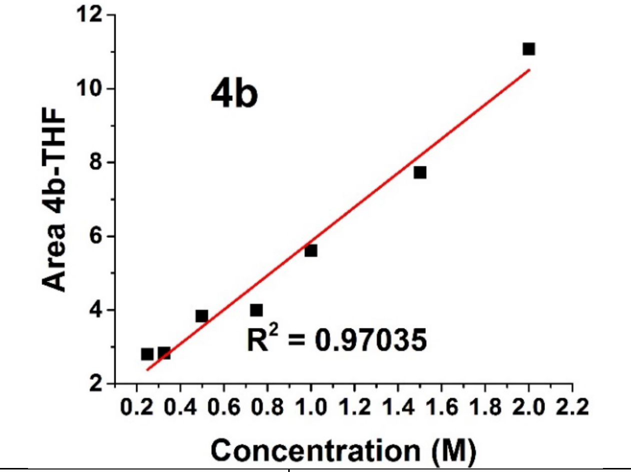

Concentration / M	Area*
2	11.0794
1.5	7.72818
1	5.61407
0.75	3.99873
0.50	3.84246
0.325	2.83919
0.25	2.79694

Supplementary Table 10. Calibration curve of the starting material **4c**, calculated processing IR spectra of this compound as a THF solution at different concentrations.

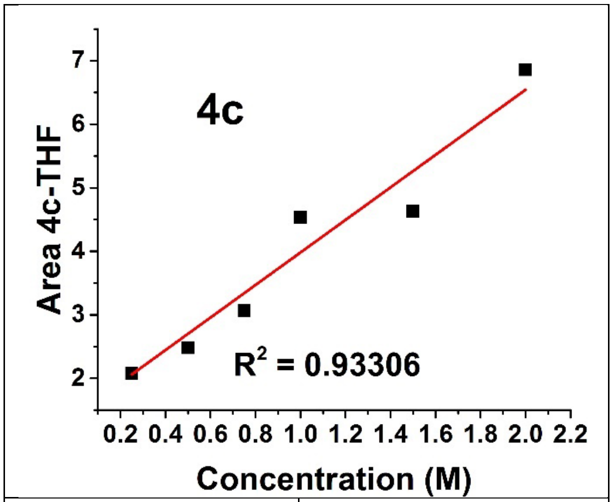

Concentration / M	Area*
2	6.85904
1.5	4.63136
1	4.53018
0.75	3.0693
0.50	2.48284
0.25	2.08193

Supplementary Table 11. Calibration curve of the starting material **4d**, calculated processing IR spectra of this compound as a THF solution at different concentrations.

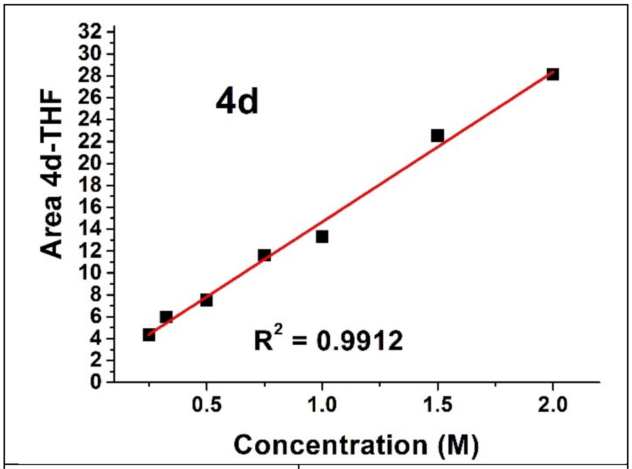

Concentration / M	Area*
2	28.11051
1.5	22.54928
1	13.30631
0.75	11.58336
0.50	7.52753
0.325	5.96645
0.25	4.33937

Supplementary Table 12. Calibration curve of the starting material **7a**, calculated processing IR spectra of this compound as a THF solution at different concentrations.

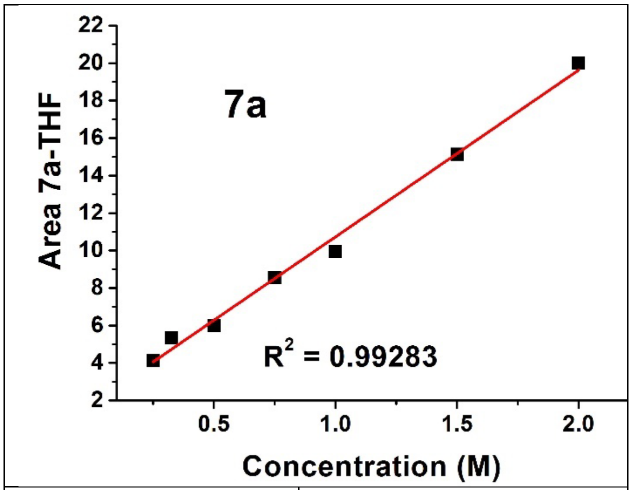

Concentration / M	Area*
2	19.98989
1.5	15.13407
1	9.95711
0.75	8.53331
0.50	6.00373
0.325	5.34107
0.25	4.14085

Supplementary Table 13. Calibration curve of the starting material **7b**, calculated processing IR spectra of this compound as a THF solution at different concentrations.

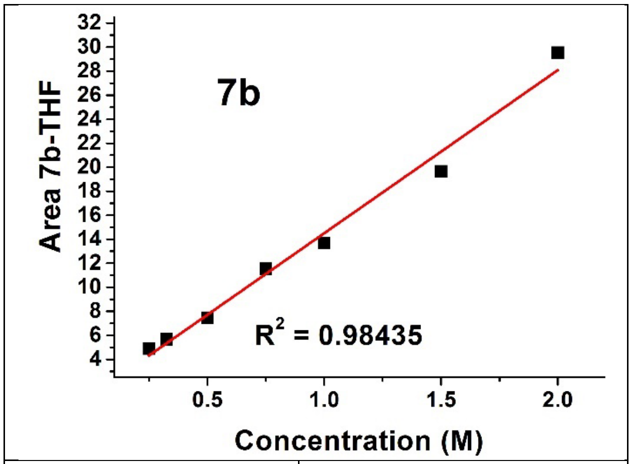

Concentration / M	Area*
2	29.52788
1.5	19.66466
1	13.6713
0.75	11.53326
0.50	7.45029
0.325	5.67297
0.25	4.89943

Supplementary Table 14. Calibration curve of the starting material **7c**, calculated processing IR spectra of this compound as a THF solution at different concentrations.

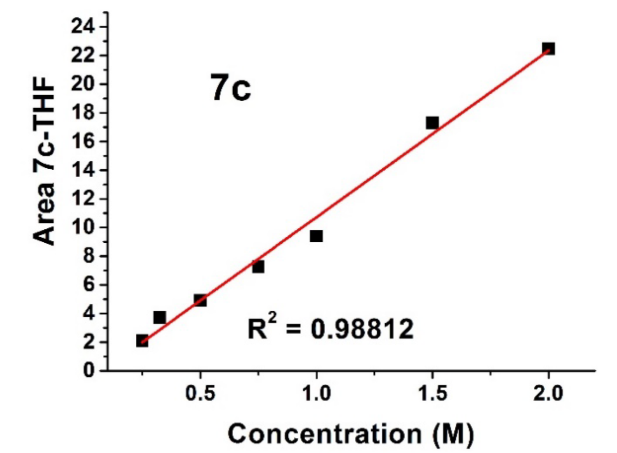

Concentration / M	Area*
2	22.47175
1.5	17.30849
1	9.4017
0.75	7.25688
0.50	4.90136
0.325	3.72284
0.25	2.10386

Supplementary Table 15. Calibration curve of the starting material **7d**, calculated processing IR spectra of this compound as a THF solution at different concentrations.

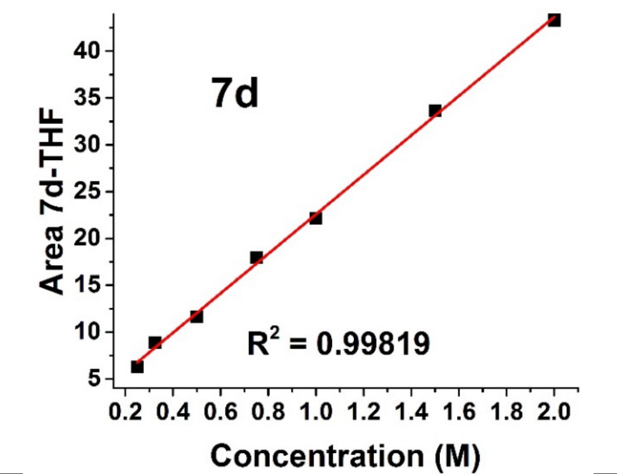

Concentration / M	Area*
2	43.30922
1.5	33.63956
1	22.15369
0.75	17.95315
0.50	11.61244
0.325	8.84256
0.25	6.2441

Supplementary Table 16. Table corresponding to the txt file loaded in the VI for the automated calculation of the correction factors. Where **b** is the slope value; **a** is the intercept value; $\mathbf{A_0}$ and $\mathbf{c_0}$ are the values of the area of the IR spectra between 1900 cm^{-1} and 650 cm^{-1} at a known concentration; **c** is the value of the expected starting material concentration if no reaction took place.

**Compounds**	**Intercept (a)**	**Slope (b)**	$\mathbf{A_0}$	$\mathbf{C_0}$ **/ M**	**C I generation / M**	**C II generation / M**	**C III generation / M**
**1**	-0.45	1.27	0.52	0.75	1.5	0.75	0.325
**2a**	-0.67	7.21	4.45	0.75	1.5	0.75	0.325
**2b**	0.96	11.89	9.92	0.75	1.5	0.75	0.325
**2c**	0.95	9.84	8.15	0.75	1.5	0.75	0.325
**2d**	-0.20	6.85	5.28	0.75	1.5	0.75	0.325
**4a**	0.71	12.67	10.65	0.75	0	0.75	0.325
**4b**	1.22	4.64	3.99	0.75	0	0.75	0.325
**4c**	1.42	2.56	3.07	0.75	0	0.75	0.325
**4d**	0.97	13.69	11.58	0.75	0	0.75	0.325
**7a**	1.83	8.89	8.53	0.75	0	0	0.325
**7b**	0.93	13.58	11.53	0.75	0	0	0.325
**7c**	-0.91	11.63	7.26	0.75	0	0	0.325
**7d**	1.48	21.09	17.95	0.75	0	0	0.325

Supplementary Table 17. The solution used for the navigation of the two generation reaction mixtures in flow.

Pump number	Compound	Concentration (M)	Solution (mL)	mmol	g	mL
1	1	3	10	30	1.984	2.522
	0.5 mol% $Sc(OTf)_3$ in 10 mL is 0.15 mmol, 74 mg					
2	2a	3	10	30	2.102	2.474
3	2b	3	10	30	2.944	3.424
4	2c	3	10	30	2.524	2.934
5	2d	3	10	30	2.945	3.106
6	4a	1.5	5	7.5	0.699	0.684
7	4b	1.5	5	7.5	0.909	0.967
8	4c	1.5	5	7.5	0.744	0.853
9	4d	1.5	5	7.5	0.909	0.951
14	Reservoir of pure THF					

Supplementary Table 18. The pathways explored and their MSE and RSI values for the two generation reaction mixtures in flow.

Reaction	Pump combination	Pathway combination	MSE (10^{-2})	RSI (%)
1	1-2	1+2a	1.238	93.08
2	1-3	1+2b	0.036	2.70
3	1-4	1+2c	0.043	3.24
4	1-5	1+2d	0.013	0.98
5	1-2-6	1+2a+4a	0.596	18.60
6	1-2-7	1+2a+4b	0.804	25.10
7	1-2-8	1+2a+4c	1.657	51.70
8	1-2-9	1+2a+4d	0.148	4.60

Supplementary Table 19. The solution used for the navigation of the three generation reaction mixtures in flow.

Pump number (Px)	Compound	Concentration (M)	Solution (mL)	mmol	g	mL
1	1	3	20	60	3.968	5.044
	0.5 mol% $Sc(OTf)_3$ in 20 mL is 0.3 mmol, 158 mg					
2	2a	3	20	60	4.204	4.968
3	2b	3	20	60	5.888	6.848
4	2c	3	20	60	5.048	5.868
5	2d	3	20	60	5.889	6.212
6	4a	1.5	10	15	1.398	1.368
	Na BH_3CN 15 mmol, 992.6 mg					
7	4b	1.5	10	15	1.818	1.934
	Na BH_3CN 15 mmol, 992.6 mg					
8	4c	1.5	10	15	1.488	1.706
	Na BH_3CN 15 mmol, 992.6 mg					
9	4d	1.5	10	15	1818	1902
	Na BH_3CN 15 mmol, 992.6 mg					
10	7a	0.75	5	3.75	0.347	0.328
11	7b	0.75	5	3.75	0.578	0.496
12	7c	0.75	5	3.75	0.527	0.435
13	7d	0.75	5	3.75	0.511	0.469
14	Reservoir of pure THF					

Supplementary Table 20. The pathways explored and their conversion, MSE and RSI values for the three generation reaction mixtures in flow.

Reaction	Pump combination	Pathway combination	MSE (10^{-2})	RSI (%)
1	1-2	1+2a	1.238	93.08
2	1-3	1+2b	0.036	2.70
3	1-4	1+2c	0.043	3.24
4	1-5	1+2d	0.013	0.98
5	1-2-6	1+2a+4a	0.085	24.70
6	1-2-7	1+2a+4b	0.080	23.07
7	1-2-8	1+2a+4c	0.118	33.97
8	1-2-9	1+2a+4d	0.063	18.26
9	1-2-8-10	1+2a+4c+7a	0.583	27.00
10	1-2-8-11	1+2a+4c+7b	0.529	24.50
11	1-2-8-12	1+2a+4c +7c	0.531	24.60
12	1-2-8-13	1+2a+4c +7d	0.517	23.90

Supplementary Figures

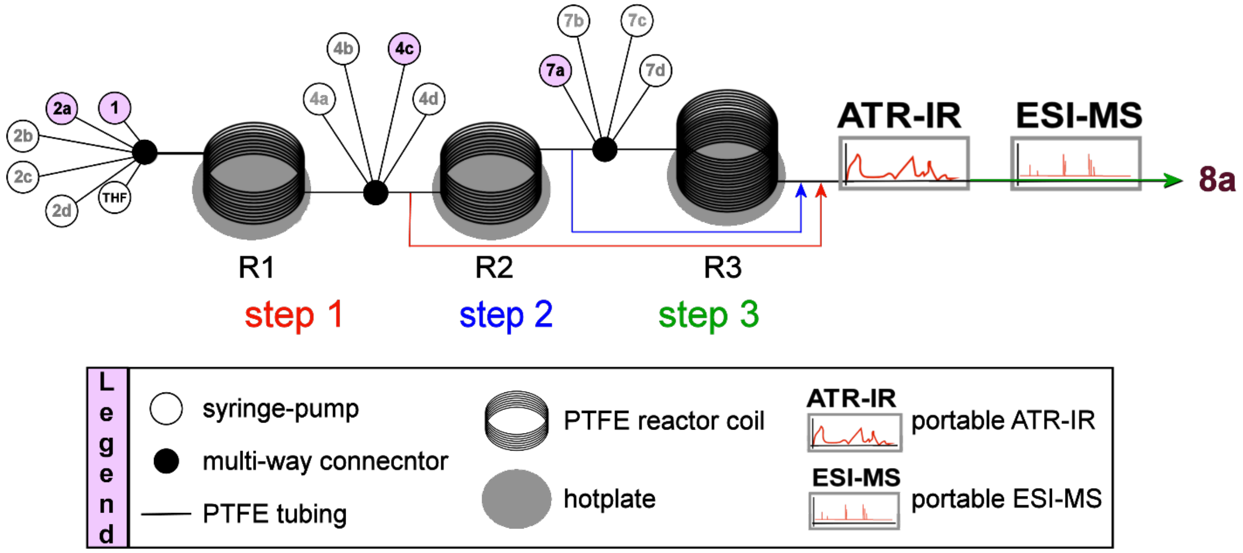

Supplementary Figure 1. Schematic of the decision making platform flow setup, for running up to three-step reactions; this setup is equipped with 14 syringe-pumps, three flow reactors and two in-line analytics connected to each other.

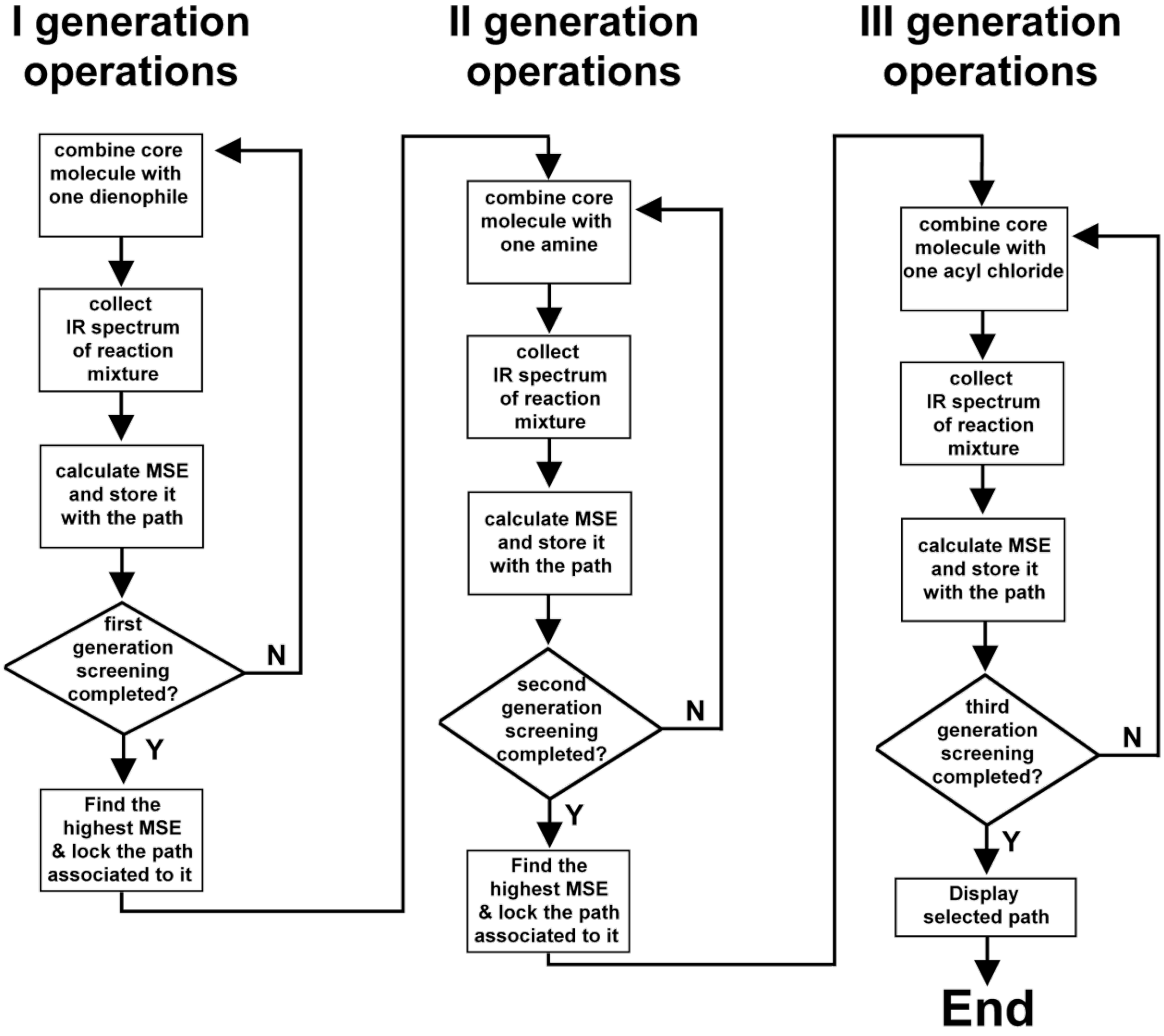

Supplementary Figure 2. Flow diagram of the navigation through different paths using the decision making platform. The algorithm of this platform is used as a reactivity navigation system and each reagent in a given generation is selected randomly.

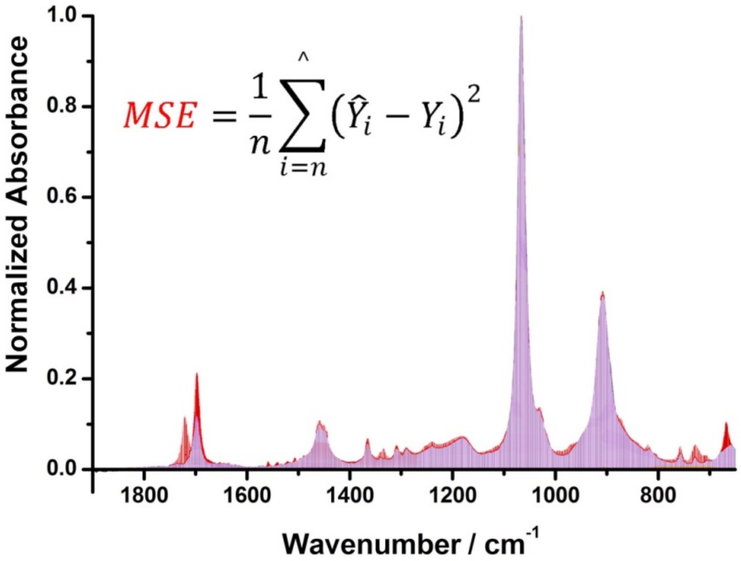

Supplementary Figure 3. A practical example with area overlap (in pink) and difference of area (in red) of the IR spectra of compound **3a** and its starting materials **1** and **2a**.

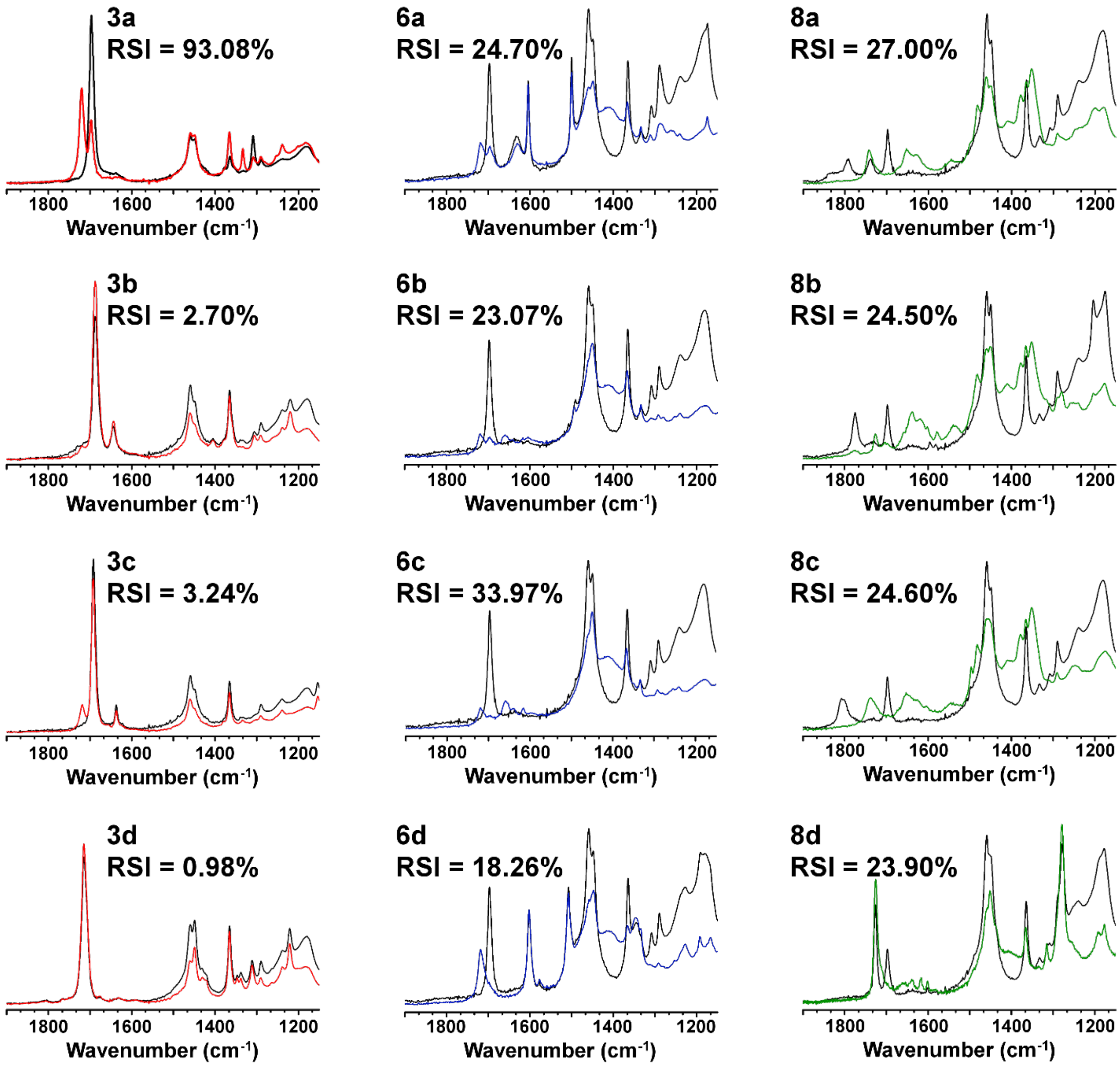

Supplementary Figure 4. Illustration of the difference between the IR spectrum of the summed starting materials (in black) and the experimental IR spectrum – 1st generation in red, 2nd generation in blue, and 3rd generation in green.

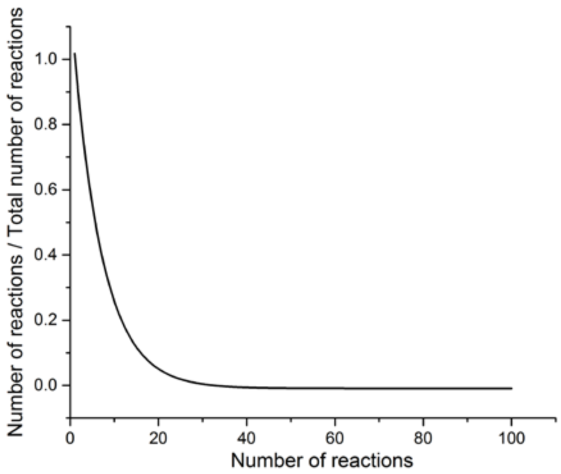

Supplementary Figure 5. Graph of the number of reactions / total number of reactions plotted against the total number of reactions for systems with the number of reagents = the number of generations between 1 and 10.

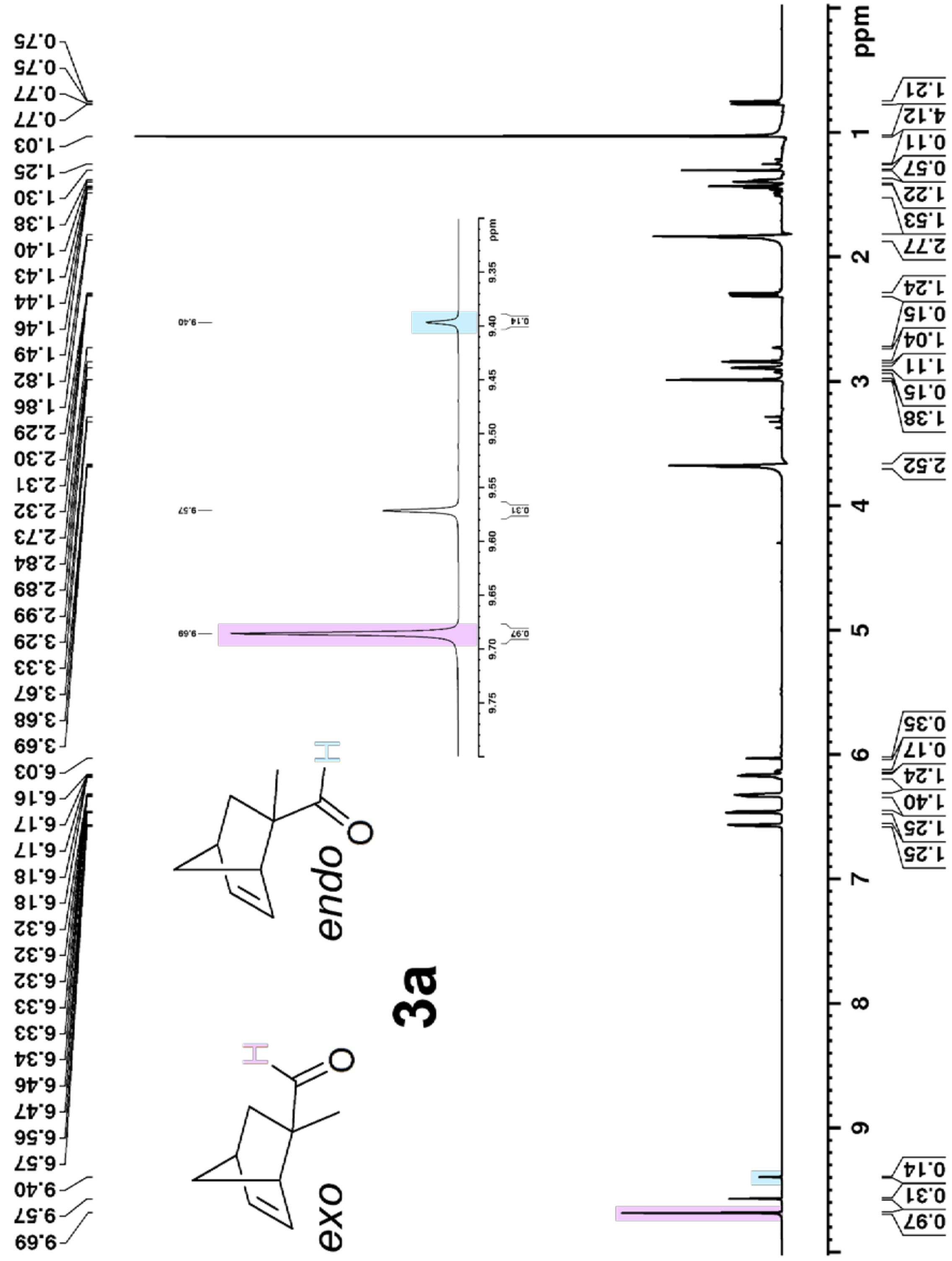

Supplementary Figure 6. ^{1}H NMR of the reaction mixture **3a**.

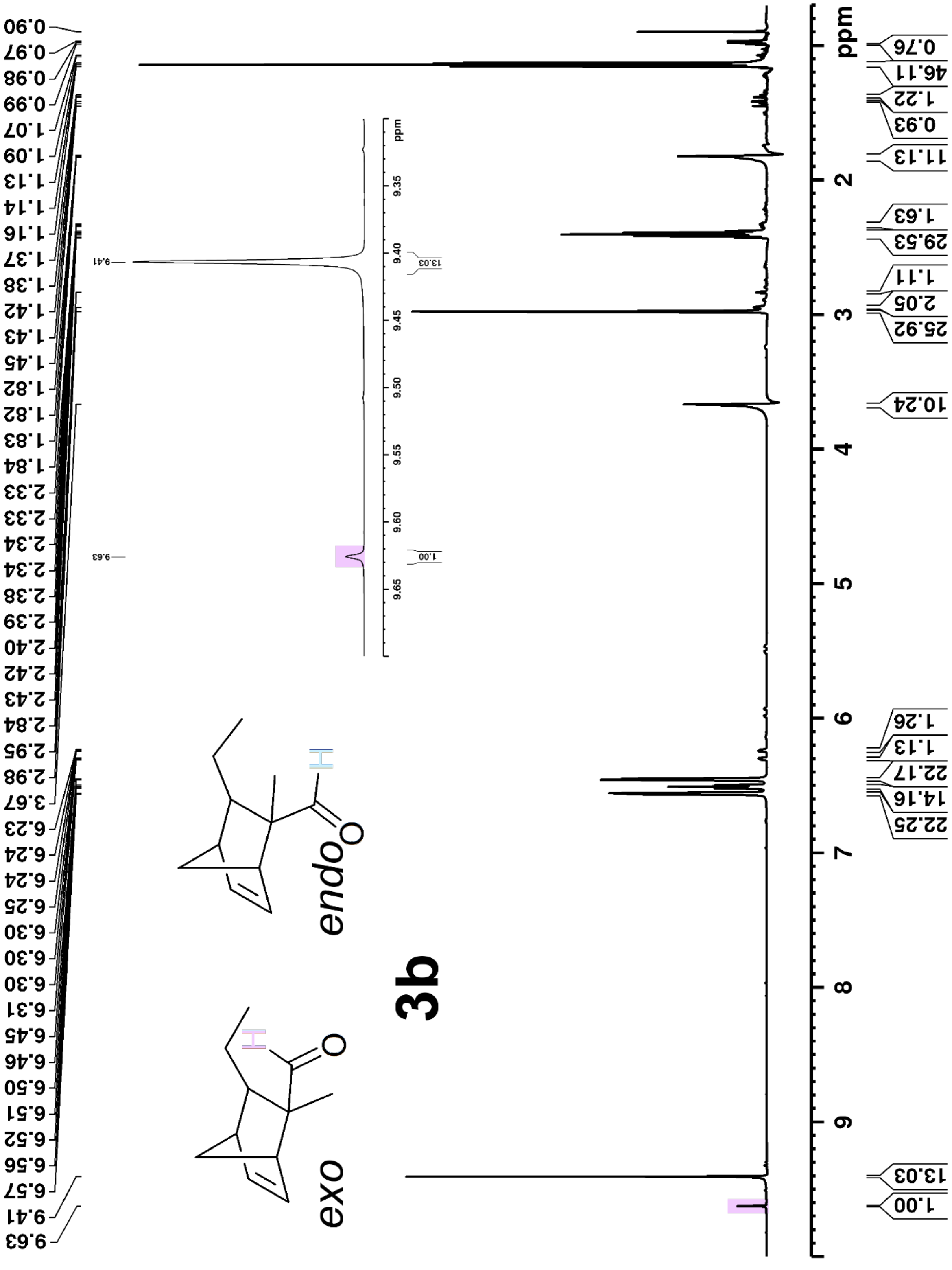

Supplementary Figure 7. ^{1}H NMR of the reaction mixture **3b**.

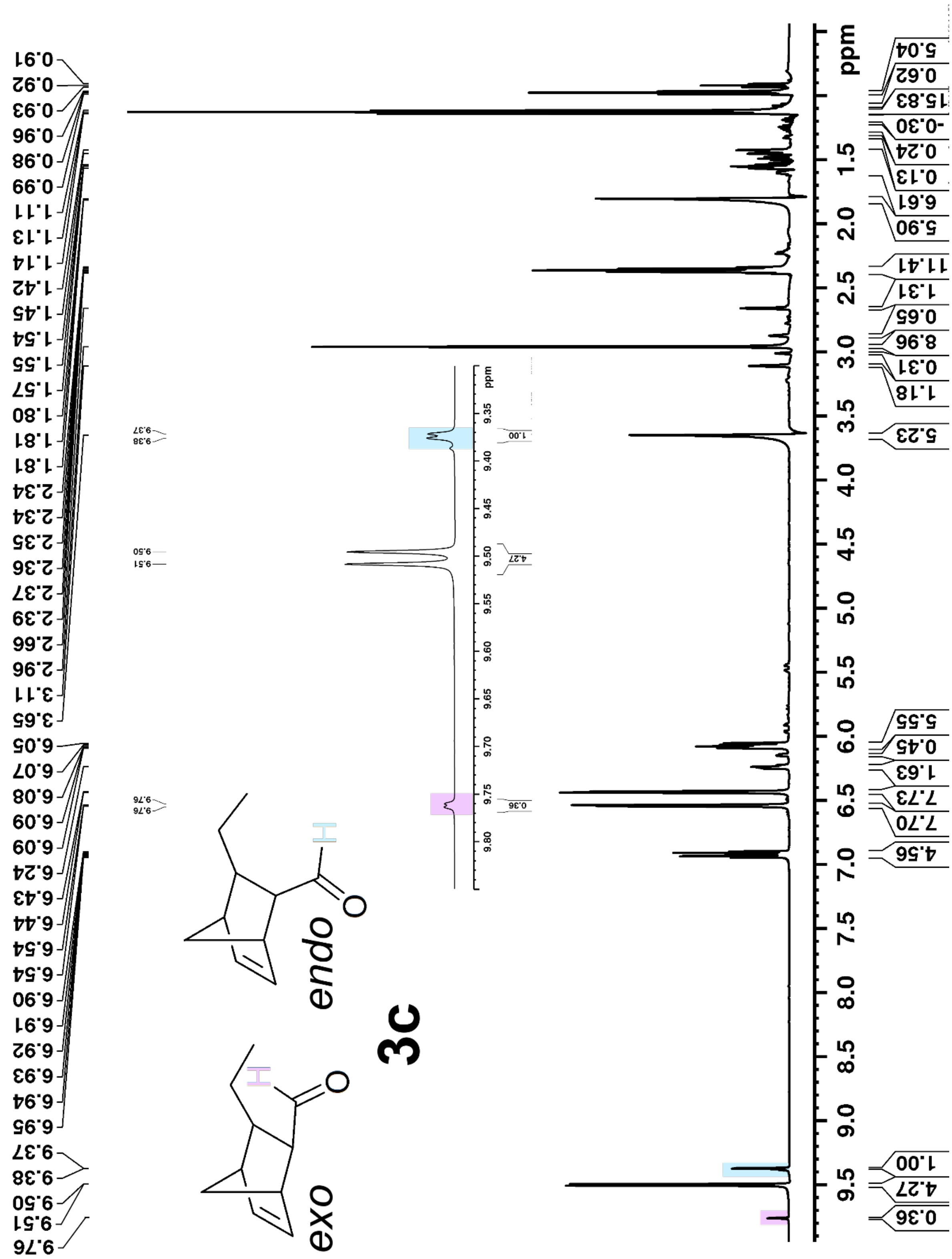

Supplementary Figure 8. ^{1}H NMR of the reaction mixture **3c**.

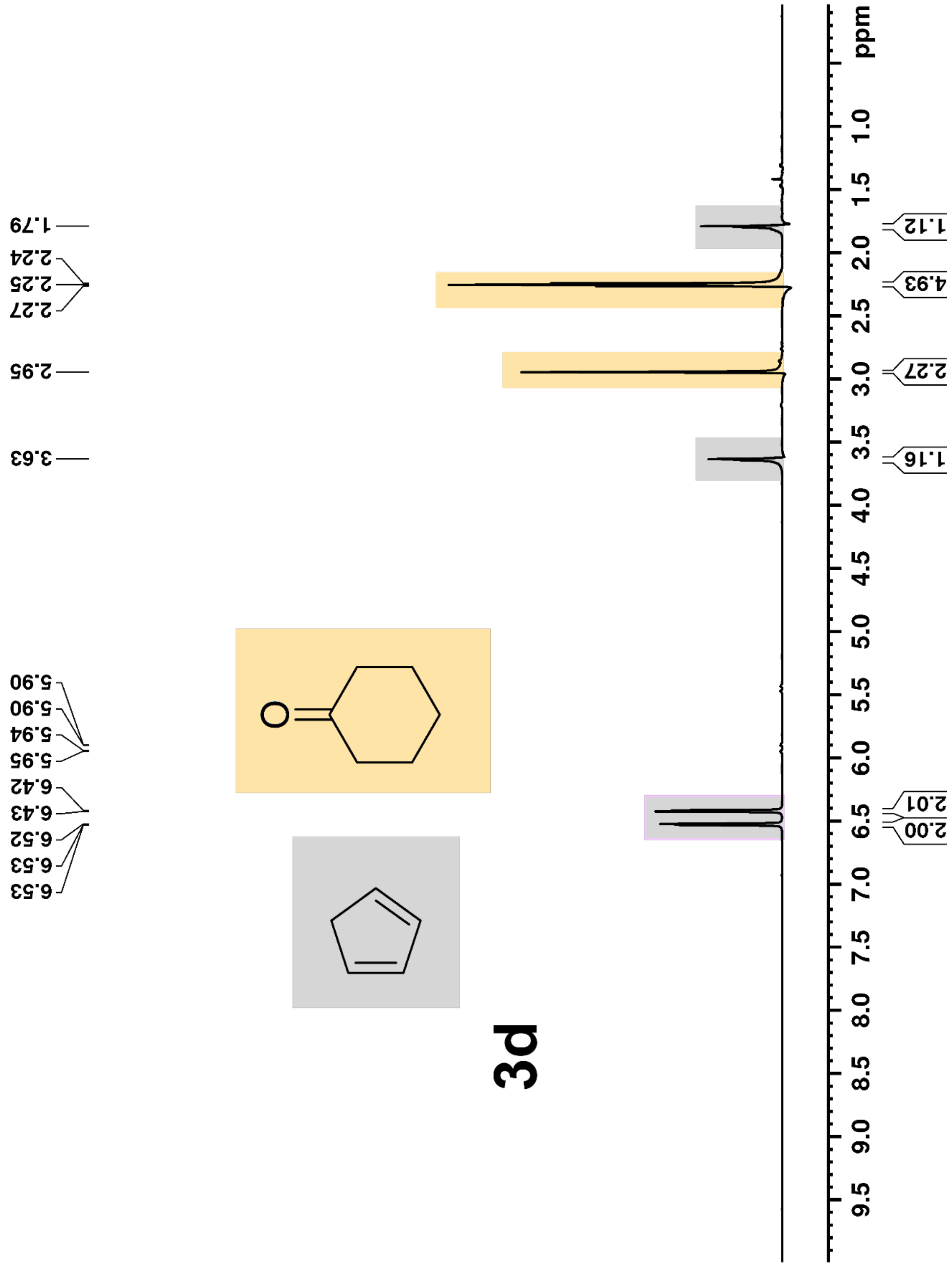

Supplementary Figure 9. ^{1}H NMR of the reaction mixture **3d**.

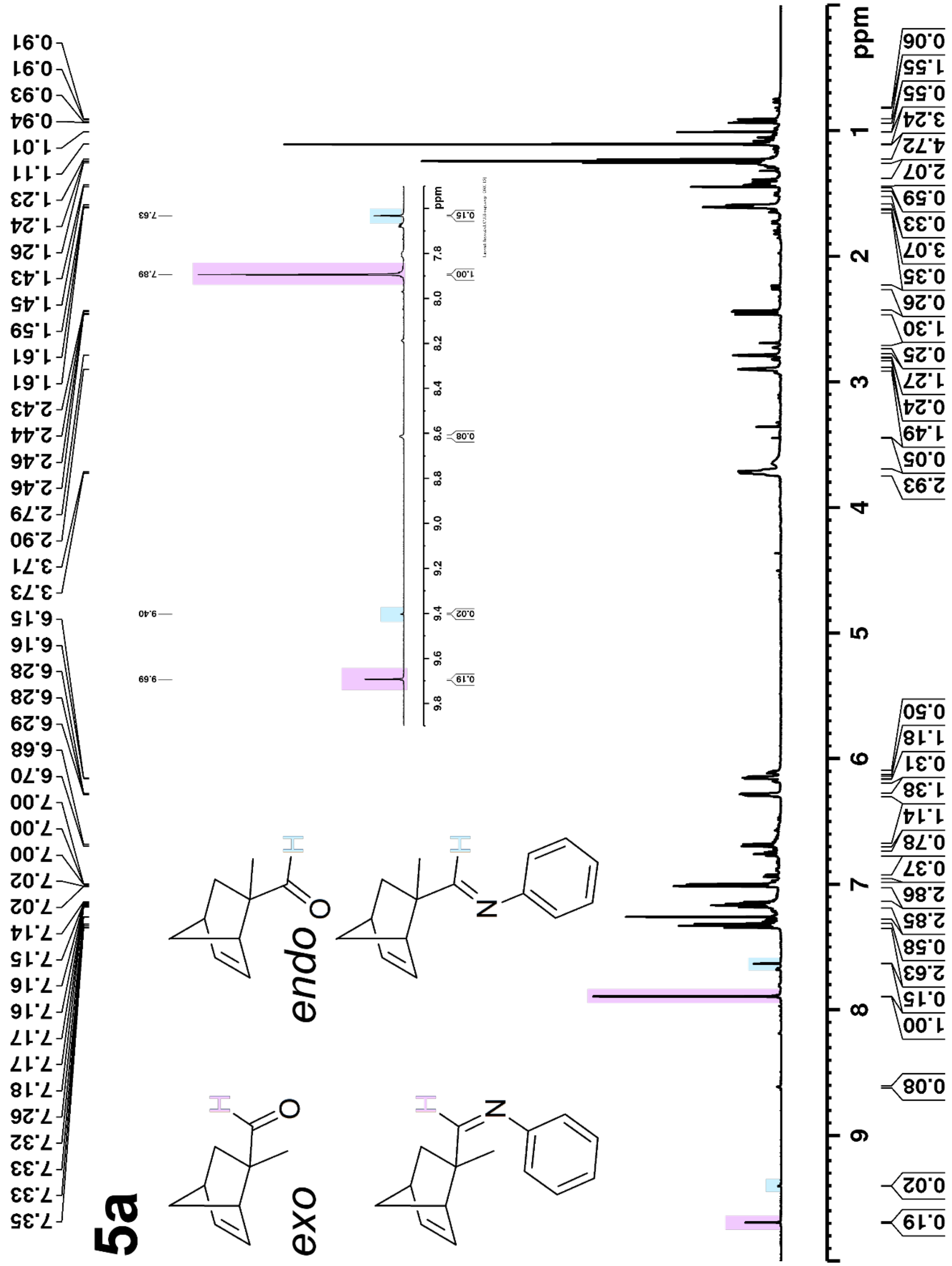

Supplementary Figure 10. ^{1}H NMR of the reaction mixture **5a**.

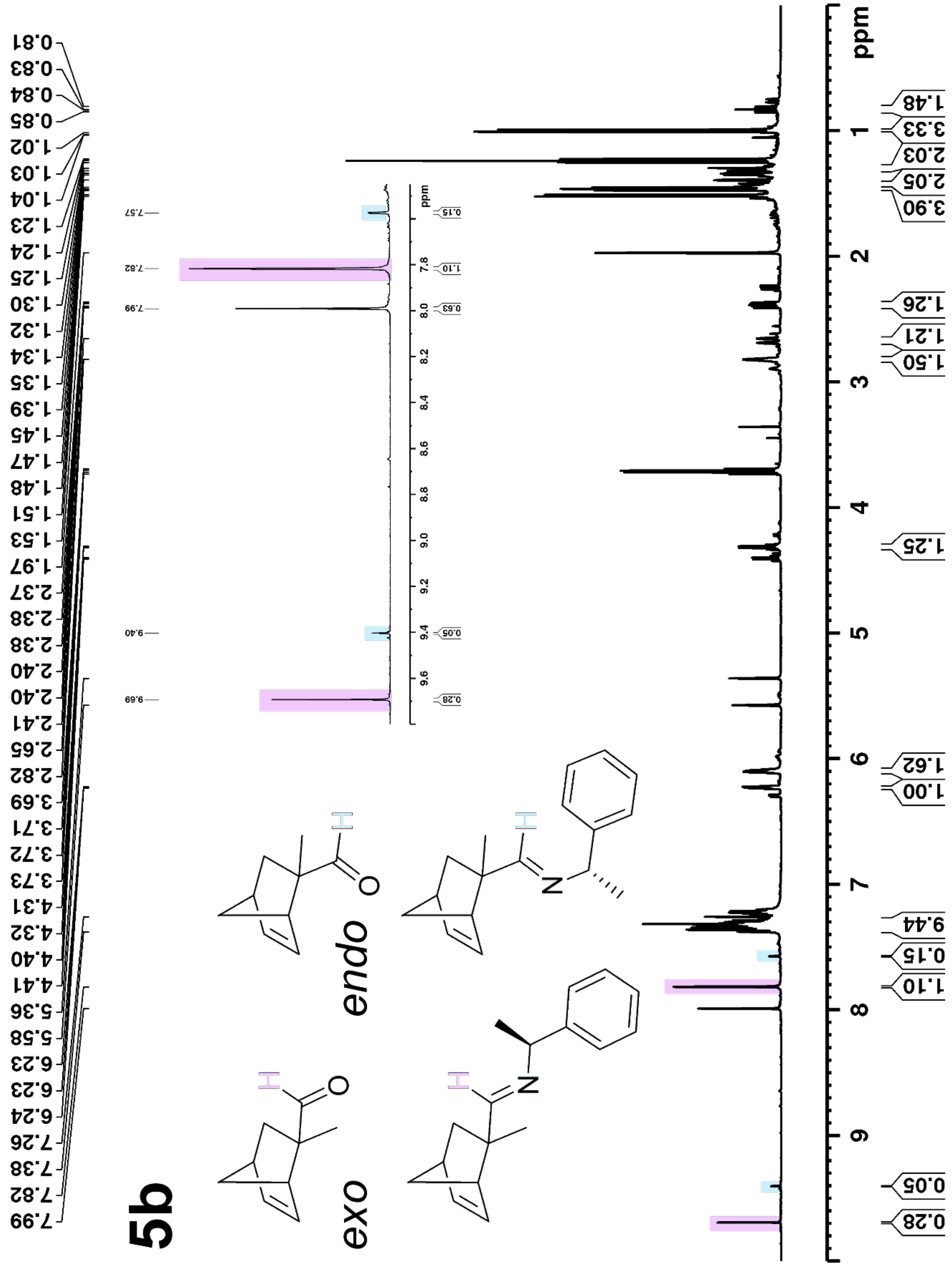

Supplementary Figure 11. ^{1}H NMR of the reaction mixture **5b**.

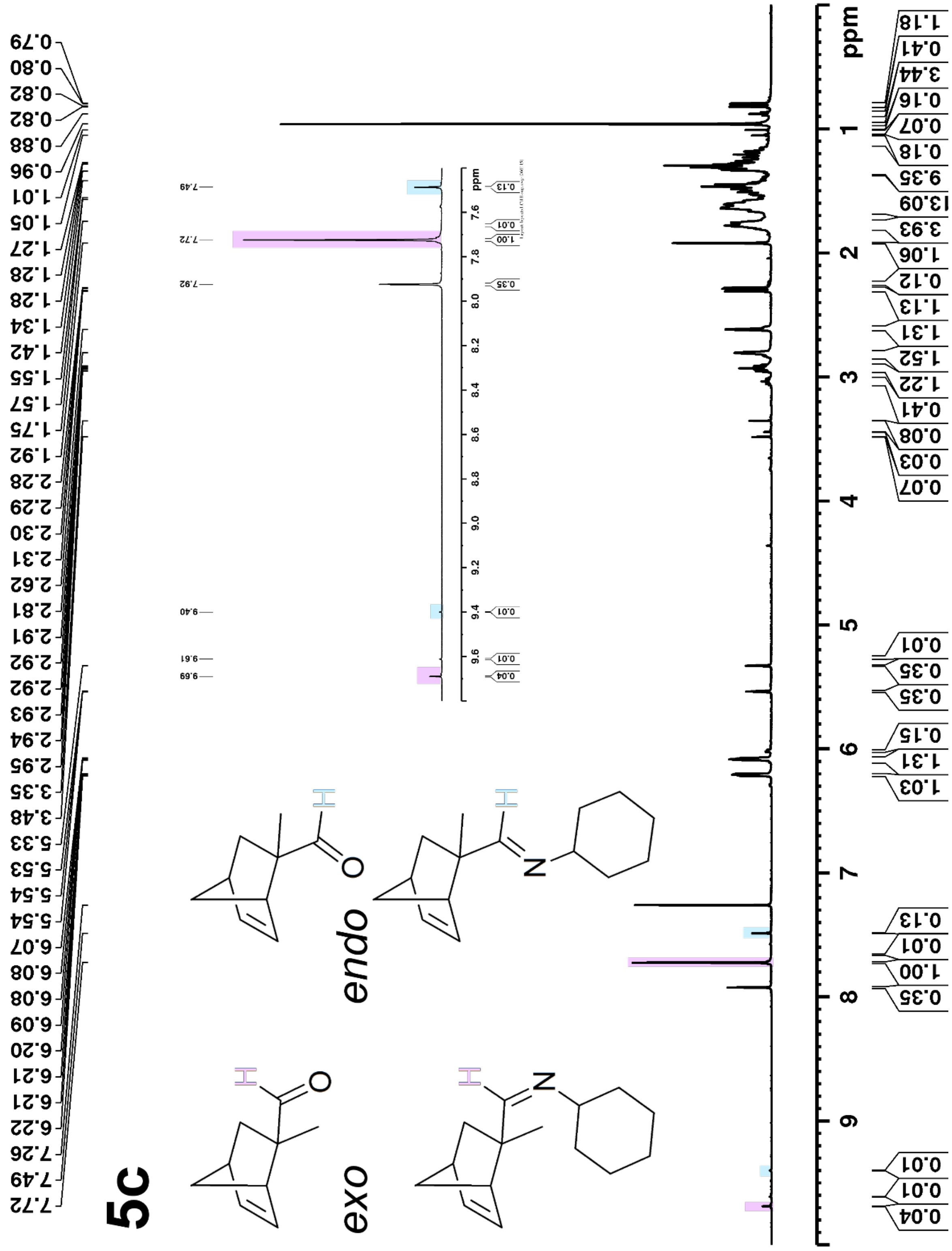

Supplementary Figure 12. ^{1}H NMR of the reaction mixture **5c**.

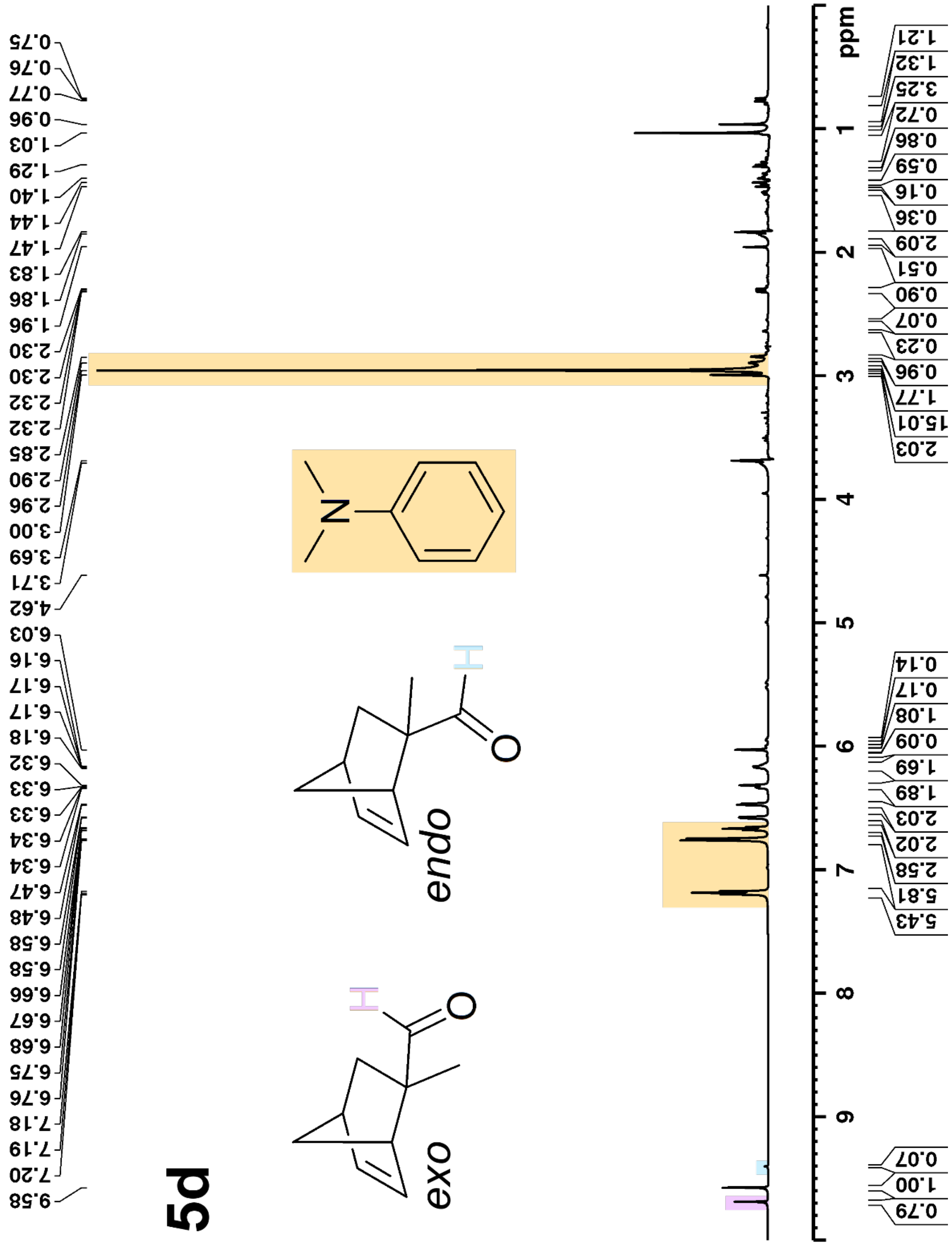

Supplementary Figure 13. ^{1}H NMR of the reaction mixture **5d**.

Supplementary Notes

Supplementary Note 1

Details on the RSI/pathway dependent algorithm

The pathway dependent chemistry algorithm screens the outcome of a core molecule reacted in a three-step reaction with four different reagents in each step. It then evaluates the reactivity of each pathway explored with the reactivity section index (RSI). A schematic of the flow diagram of this algorithm is illustrated in **Supplementary Figure 2**. When used for the navigation of the reaction network presented in this work, the outcome of each reaction step forms a progressively more populated generation to navigate. Therefore, the first, second and third generations are formed from four, sixteen and sixty-four different reactant combinations, respectively. This algorithm was developed to screen all 4 reactions in the first step, then select the one with the highest RSI, which is associated to the most reactive pathway among those screened. It then locks this selected pathway as the new core molecule to screen, in a similar way with the second set of reactants. At the end of the second generation screening, a new synthetic pathway is locked which is consequently reacted with the third set of reactants. The final decision point of this algorithm concludes with the selection of the most reactive pathway of the chemical space screened. After each reaction, the IR spectrum of the mixture is measured and the MSE is calculated. After completing the screening of a generation, the RSI are derived from the MSE and then are compared in order to select the biggest value and consequently the pathway associated with it. The same operations are repeated for the other two steps, in which three reactions are screened and compared. A core feature of this platform is the algorithm used to assess reactivity of the chemical reactions with the RSI, which is derived from the mean square error (MSE) of the difference of the ATR-IR spectra. Once the run of a generation is completed, the RSI of each screened pathway is calculated as shown in **Supplementary Equation 1**.

$$RSI_n = \frac{MSE_n}{\sum MSE_{gen}} \quad (1)$$

The MSE permits to estimate the difference between the IR spectra of a known combination of starting materials and the same reacted (under specific conditions). This value is defined as the mean of the squares of the difference between the actual observations – in this case an

experimental IR spectrum – and those predicted – in this case, the sum of the starting material IR spectra. The MSE equation is given below (**Supplementary Equation 2**), in which n is the number of points, $\hat{Y}$ is the vector predicted and Y is the observed vector.

$$MSE = \frac{1}{n}\sum_{i=n}^{\wedge}\left(\hat{Y}_i - Y_i\right)^2 \quad (2)$$

This value is defined as the mean of the squares of the difference between the actual observations – in this case an experimental IR spectrum – and those predicted – in this case, the sum of the starting material IR spectra (**Supplementary Figure 3**).

The spectra compared were the experimental IR associated to a specific reaction mixture and IR of the sum of the starting materials that generated that reaction mixture. The reagent spectra to sum were firstly collected, and then normalized using the solvent signal at 1066 cm^{-1} as internal standard. The starting material spectra are then corrected in terms of intensity using calibration curves, in order to match their expected concentrations in the reaction mixture if no reaction occurs and then are summed. These correction factors are automatically calculated by the algorithm using the values obtained from the calibration curves made from each starting materials. The correction factors were calculated using the equations from the calibration curves of each reagent and the calibration curves were obtained by measuring the IR spectra of the reagents at different known concentrations, and then calculating the concentration by integrating the area under the curve. The data analysis for the deconvolution and peak-area calculations of the IR spectra, and the calculation of the calibration curves, were done using Origin®. Here the deconvolution of the peaks in a selected region of the IR was obtained using by fitting multiple peaks and the calculation of the area of selected IR spectra peaks done using the integration function. Finally, the calibration curve equations were calculated using the linear fitting feature. The calibration curves and the details used to calculate them are reported for each of the starting materials is given in **Supplementary Tables 3-15**. The IR of each compound at different concentrations was normalized and then used to calculate each compound concentration. The equations used to calculate the correction factor (F) at each different reaction step are given below. These were validated and applied for each of the starting materials in the experiment as shown in **Supplementary Equations 3-5**.

$$F = \frac{(A-a)}{(A_0-a)} \quad (3)$$

$$F = A_0 + b(c - c_0) \quad (4)$$

$$F = \frac{((b\times(c-c_0)+A_0)-a)}{(A_0-a)} \quad (5)$$

Where **F** is the correction factor; **b** is the slope value; **a** is the intercept value; $\mathbf{A_0}$ and $\mathbf{c_0}$ are the values of the area of the IR spectra between 1900 cm^{-1} and 1000 cm^{-1} at a known concentration; **c** is the value of the expected starting material concentration if no reaction took place. All the necessary variables required to determine the correction factors of each starting material at each of the different reaction conditions were calculated by using the values reported in **Supplementary Table 16**, which are uploaded prior to the start of the experiment. After calculating the correction factors for each of the reactants involved in the experiments, an IR spectrum was generated as the sum of the corrected spectra and then normalized to compare to the experimental and normalized spectra, in order to calculate the value of the MSE for each exact experiment, as the difference between the area of one spectrum to the other.

Supplementary Note 2

Summary of the operations executed with VI-4 for each sequence-run of the navigation up to the second generation.

Experimental operations:

1. Collect IR background and set the reactor temperature.
2. Chose the variables.
3. Move 1.5 mL of reagents associated with P1 and P2, P3, P4 or P5 – randomly selected and excluding the combination already run – at 0.075 mL min^{-1}.
4. Wait until P1 has been completely emptied (calculated dividing the volume in P1 by the flow rate of P1).
5. Move 1.5 mL of reagent associated with P14 at a flow rate of 0.15 mL min^{-1}.
6. When P14 is half emptied (calculated dividing half volume of the reagent in P8 by the flow rate with which P14 is moved), collect IR spectrum and compare with the starting

material of this reaction combination in order to calculate the MSE value associated to this reaction.

7. Wait until P14 has been completely emptied (calculated dividing half volume of the reagent in P14 by the flow rate of P14).
8. Repeat operations 3, 4, 5, 6, and 7 in that order, trice.
9. At the end of the last experiment of the I generation, calculate the RSI values associated to each reaction mixture.
10. Compare the three RSI values in order to select the biggest one and lock the reaction path associated with it (saved as combination of pumps).
11. Move 1.125 mL of reagents associated with the pumps locked at a flow rate of 0.0325 mL min^{-1}.
12. Wait for the residence time in the first reactor (calculated dividing the volume of the first reactor by the sum of the flow rate of the two syringe pumps locked).
13. Move 0.75 mL of reagents associated with P6, P7, P8 or P9 – randomly selected and excluding the combination already run – at a flow rate of 0.075 mL min^{-1}.
14. Repeat operations 5, 6 and 7.
15. Repeat operations 11, 12, 13 and 14 in that order, trice.
16. At the end of the last experiment of the II generation, calculate the RSI values associated to each reaction mixture.
17. Compare the last three RSI values in order to select the biggest one and select the reaction path associated with it (saved as combination of pumps).

Supplementary Note 3

*Summary of the operations executed with VI-4 for each sequence-run of the navigation up to the third generation.*Experimental operations:

1. Collect IR background and set the reactor temperature.
2. Chose the variables.
3. Move 3 mL of reagents associated with P1 and P2, P3, P4 or P5 – randomly selected and excluding the combination already run – at 0.15 mL min^{-1}.

4. Wait until P1 has been completely emptied (calculated dividing the volume in P1 by the flow rate of P1).
5. Move 1.5 mL of reagent associated with P14 at a flow rate of 0.3 mL min^{-1}.
6. When P14 is half emptied (calculated dividing half volume of the reagent in P14 by the flow rate with which P14 is moved), collect IR spectrum and compare with the starting material of this reaction combination in order to calculate the MSE value associated to this reaction.
7. Wait until P14 has been completely emptied (calculated dividing half volume of the reagent in P14 by the flow rate of P14).
8. Repeat operations 3, 4, 5, 6, and 7 in that order, trice.
9. Compare the three RSI values in order to select the biggest one and lock the reaction path associated with it (saved as combination of pumps).
10. Move 1.125 mL of reagents associated with the pumps locked at a flow rate of 0.0325 mL min^{-1}.
11. Wait for the residence time in the first reactor (calculated dividing the volume of the first reactor by the sum of the flow rate of the two syringe pumps locked).
12. Move 0.75 mL of reagents associated with P6, P7, P8 or P9 – randomly selected and excluding the combination already run – at a flow rate of 0.075 mL min^{-1}.
13. Repeat operation 4.
14. Move 4.5 mL of reagent associated with P14 at a flow rate of 0.15 mL min^{-1}.
15. Repeat operations 6, 7 and then trice operations 10, 11, 12, 4, 14, 6 and 7 in that order.
16. Compare the last three RSI values in order to select the biggest one and lock the reaction path associated with it (saved as combination of pumps).
17. Move 1.5 mL of reagents associated with the pumps locked at a flow rate of 0.0325 mL min^{-1}.
18. Wait for the residence time in the first reactor (calculated dividing the volume of the first reactor by the sum of the flow rate of the first two syringe pumps locked).
19. Move 0.75 mL of reagents associated with the pump locked in the second step of the reaction at a flow rate of 0.075 mL min^{-1}.
20. Wait for the residence time in the second reactor (calculated dividing the volume of the second reactor by the sum of the flow rate of the three syringe pumps locked).

21. Move 0.75 mL of reagents associated with P10, P11, P12 or P13 – randomly selected and excluding the combination already run – at 0.15 mL min^{-1}.
22. Repeat operation 4.
23. Move 1.5 mL of reagent associated with P14 at a flow rate of 0.3 mL min^{-1}.
24. Repeat operations 6 and 7, and then trice operations 17, 18, 19, 20, 21, 4, 23, 6 and 7 in that order.
25. Compare the last three RSI values in order to select the biggest one and lock the reaction path associated with it (saved as combination of pumps).

Supplementary Note 4

Characterization of the compounds formed in the reaction pathway navigated; e.g. compounds 3a, 3b, 3c, 5a, 5b, 5c, 6a, 6b, 6c, 8a, 8b, 8c.

All the reaction pathways navigated in the first two generations were analyzed by at-line ^{1}H NMR as mixtures. The reactivity of the pathways was measured in all cases using the aldehyde peaks of the starting materials. In particular:

- For the I generation the aldehyde peak of the starting material was compared with the new aldehyde peaks associated to the formation of the Diesl-Alder product **3** as *endo*/*exo* mixture (See **Supplementary Figures 6-9**).
- For the II generation forming imines **5**, the conversion of the selected cycloadduct **3** was calculated using the integrals of the aldehyde and imine peaks (See **Supplementary Figures 10-13**).
- For the II generation forming secondary amines **6**, the reactivity of compound **3a** was measured by a more complex calculation: first the integrals of the residual aldehyde peaks at 9.40 (**3a** endo), 9.69 (**3a** exo) and 9.57 (**2a**) ppm were normalized to the intensity of the methanol peak at 2.99 ppm in each of the four spectra. Then the sum of these normalized integrals was calculated for each of the four spectra. The value corresponding to compound **6c** was 0.00, so it means that the 100% of aldehyde reacted. The value corresponding to the reaction mixture obtained from the reaction of **3a** with **2d** was 1.27 and it corresponded to the 0% of aldehydes reactivity. The value calculated for the reaction between **3a** and **2a**

was 0.73 which corresponded to the 42.5% of **3a** reacted. The value calculated for the reaction between **3a** and **2b** was 0.15 which corresponded to the 88.2% of **3b** reacted.

Compounds identified in the reaction mixtures by at-line 1H NMR analyses.

2-methyl-bicyclo[2.2.1]hept-5-ene-2-carbaldehyde (3a)

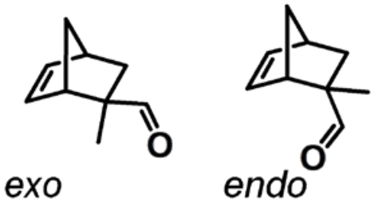

Conversion 77 % calculated by ^{1}H NMR comparing the protons of the aldehydes; ratio *endo*:*exo* isomers 22:78.

MS **[**ESI^{+}]: a peak was observed with m/z 137.3 and was assigned as $C_9H_{13}O^+$ ([M+H]+ predicted m/z 137.1).

[GC]: peaks were observed with *m/z* 136.1 at 10.41 and 10.45 min and were assigned as $C_9H_{12}O$ ($[M]^+$ predicted *m/z* 136.1).

^{1}H NMR [$CDCl_3$, 500 MHz, 20 °C]: (minor product *endo*) δ 9.40 (s, 1H, C*H*O), 6.18 (dd, 1H, *J=5.7 Hz, J= 3.1 Hz*), 6.13 (dd, 1H, *J=5.7 Hz, J= 3.0 Hz*), 1.87-1.83 (m, 2H), 1.67-1.64 (m, 3H), 1.30 (s, 3H), 0.78-0.74 (m, 1H *endo*, 1H *exo*); (major product *exo*) δ 9.69 (s, 1H, C*H*O), 6.33 (dd, 1H, *J=5.6 Hz, J=3.0 Hz*), 6.17 (dd, 1H, *J=5.6 Hz, J=3.0 Hz*), 2.30 (dd, 1H, *J=3.8 Hz, J=12.0 Hz*), 1.52-1.43 (m, 1H), 1.40-1.38 (m, 2H), 1.27-1.22 (m, 1H), 1.03 (s, 3H), 0.78-0.74 (m, 1H *endo*, 1H *exo*).

^{13}C NMR [$CDCl_3$, 125 MHz, 20 °C]: (major + minor products) δ 20.1 (CH_3), 34.7 (CH_2), 43.2 (CH), 47.7 (CH_2), 48.5 (CH), 53.9 (C_q), 133.1 (HC=CH, major), 135.2 (HC=CH, minor), 137.5 (HC=CH, minor), 139.6 (HC=CH, major), 205.8 (C-aldehyde major), 206.3 (C-aldehyde minor) ppm.

IR [ATR]: the signal assigned as the C=O moiety of the starting material (peak at 1696 cm^{-1}) decreased; a new signal assigned as the C=O moiety of the new aldehyde appeared at 1720 cm^{-1}.

2-methyl-3-ethyl-bicyclo[2.2.1]hept-5-ene-2-carbaldehyde (3b)

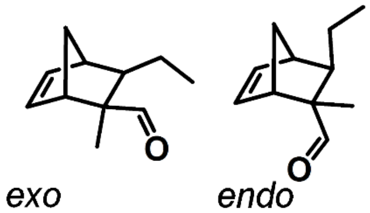

Conversion 7 % calculated by ^{1}H NMR comparing the protons of the aldehydes; ratio *endo*:*exo* isomers 0:100.

MS [GC]: a peak was observed with *m/z* 164.1 at 13.3 and was assigned as $C_{12}H_{16}O$ ($[M]^+$ predicted *m/z* 164.1).

^{1}H NMR [$CDCl_3$, 500 MHz, 20 °C]: (only one product, *exo*): δ 9.63 (s, 1H, C*H*O), 6.30 (dd, 1H, *J=5.6 Hz, J=3.0 Hz*), 6.24 (dd, 1H, *J=5.6 Hz, J=3.0 Hz*), 2.94 (bb, 2H), 2.84 (bb, 1H), 2.35-2.32 (m, 1H), 1.42 (bb, 1H), 1.38-1.37 (m, 3H), 0.99 (bb, 1H), 0.98-0.96 (m, 3H), 0.90 (s, 3H).

^{13}C NMR [$CDCl_3$, 125 MHz, 20 °C]: (*exo*) δ 13.3 (CH_3), 15.2 (CH_3), 22.7 (CH_2), 45.0 (CH), 45.9 (CH), 46.0 (CH_2), 50.3 (CH), 53.4 (C_q), 135.2 (HC=CH), 137.4 (HC=CH), 205.9 (C-aldehyde) ppm.

IR [ATR]: the signal assigned as the C=O moiety of the starting material (peak at 1687 cm^{-1}) decreased; a new signal assigned as the C=O moiety of the new aldehyde appeared at 1719 cm^{-1}.

3-ethyl-bicyclo[2.2.1]hept-5-ene-2-carbaldehyde (3c)

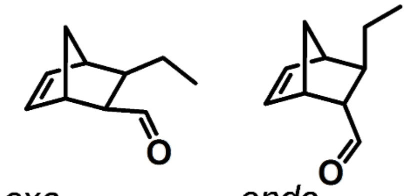

Conversion 20 % calculated by ^{1}H NMR comparing the protons of the aldehydes; ratio *endo*:*exo* isomers 73:27.

MS **[**GC]: peaks were observed with *m/z* 150.1 at 12.4 and 12.9 min and were assigned as $C_{10}H_{14}O$ ($[M]^+$ predicted *m/z* 150.1).

^{1}H NMR [$CDCl_3$, 500 MHz, 20 °C]: (major product *endo*) δ 9.37 (d, 1H, *J*=3.3 *Hz*, C*H*O), 6.24 (dd, 1H, *J*=5.7 *Hz, J*=3.2 *Hz*), 6.08 (dd, 1H, *J*=5.7 *Hz*, *J*=3.2 *Hz*), 3.10 (bb, 1H), 2.66 (bb, 1H), 1.63-1.41 (m, 6H), 0.98 (t, 3H, *J*=7.3 *Hz*); (minor product *exo*) δ 9.76 (d, 1H, *J*=2.8 *Hz*, C*H*O), 6.23 (dd, 1H, *J*=5.8 *Hz, J*=2.9 *Hz*), 6.14 (dd, 1H, *J*=5.8 *Hz, J*=3.1 *Hz*), 3.01 (bb, 1H), 2.87 (bb, 1H), 1.63-1.41 (m, 6H), 0.92 (t, 3H, *J*=7.3 *Hz*).

^{13}C NMR [$CDCl_3$, 125 MHz, 20 °C]: (major *endo*) δ 12.9 (CH_3), 28.6 (CH_2), 44.1 (CH), 45.0 (CH), 46.9 (CH_2), 47.0 (CH), 59.9 (CH), 132.9 (HC=CH), 138.8 (HC=CH), 205.1 (C-aldehyde) ppm.

IR [ATR]: the signal assigned as the C=O moiety of the starting material (peak at 1693 cm^{-1}) decreased; a new signal assigned as the C=O moiety of the new aldehyde appeared at 1719 cm^{-1}.

2-methyl-bicyclo[2.2.1]hept-5-en-2-ylmethylene-N-(phenyl)-amine (5a)

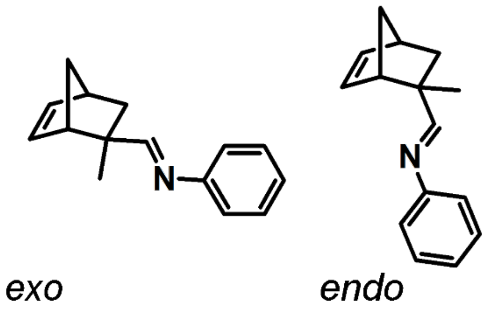

Conversion 84 % (calculated by ^{1}H NMR) including the side product obtained from the reaction of **3a** with **4a**. Ratio *endo*:*exo* isomers 13:87.

MS [ESI, positive mode]: peaks were observed with *m/z* 212.4 and 234.1 and were assigned as $C_{15}H_{18}N$ ($[M+H]^+$ predicted *m/z* 212.1) and $C_{15}H_{17}NNa$ ($[M+Na]^+$ predicted *m/z* 234.1), respectively.

[GC]: peaks were observed with *m/z* 211.1 at 20.01 and at 22.3 min and were assigned as $C_{15}H_{17}O$ ($[M]^+$ predicted *m/z* 211.4).

^{1}H NMR [$CDCl_3$, 500 MHz, 20 °C]: (major product, *exo*) δ 7.89 (s, 1H, C*H*N), 7.35-7.31 (m, 2H, *Ar*), 7.02-7.00 (m, 3H, *Ar*), 6.28 (dd, 1H, *J=3.0 Hz, J=5.6 Hz*), 6.15 (dd, 1H, *J=3.0 Hz, J=5.6 Hz*), 3.75-3.70 (m, 2H), 2.92 (bb, 1H), 2.78 (bb, 1H), 2.45 (dd, 1H, *J=3.9 Hz, J=11.7 Hz*), 1.11 (s, 3H), 0.92 (dd, 1H, *J=2.6 Hz, J=11.7 Hz*). The minor product, only a peak at 7.636 ppm can be clearly assigned to the *endo* isomer.

^{13}C NMR [$CDCl_3$, 125 MHz, 20 °C]: (major + minor products) δ 23.5 (CH_3), 36.9 (CH_2), 43.5 (CH), 47.9 (CH_2), 50.1 (CH), 58.5 (C_q), 120.6 (CH*Ar*), 125.2 (CH*Ar*), 129.0 (CH*Ar*), 133.8 (HC=CH), 138.9 (HC=CH), 152.6 (C_{qAr}), 173.4 (C-imine) ppm.

IR [ATR]: the signals assigned as the C=O moieties of compounds **2a** and **3a** (peaks at 1696 and 1720 cm^{-1}, respectively) disappeared completely. A new peak assigned as the C=N moiety of compound **5a** (and/or the corresponding side product) appeared at 1642 cm^{-1}.

2-methyl-bicyclo[2.2.1]hept-5-en-2-ylmethylene-(1-phenyl-ethyl)-amine (5b)

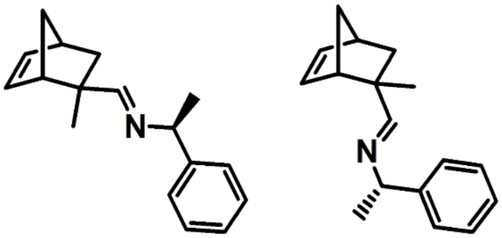

Conversion 84 % (calculated by ^{1}H NMR) including the side product obtained in significant amount from the reaction of **3a** with **4b**. Ratio *endo:exo* isomers 35:65.

MS [ESI, positive mode]: peaks were observed with *m/z* 240.1 and 262.2 and were assigned as $C_{17}H_{22}N$ ($[M+H]^+$ predicted *m/z* 240.2) and $C_{17}H_{21}NNa$ ($[M+Na]^+$ predicted *m/z* 262.2), respectively.

[GC]: peaks were observed with *m/z* 239.1 at 19.6 and at 19.7 min and were assigned as $C_{17}H_{21}O$ ($[M]^+$ predicted *m/z* 239.2).

^{1}H NMR [$CDCl_3$, 500 MHz, 20 °C]: (major + minor products) δ 7.82 (s, 1H, C*H*N major), 7.58 (s, 1H, C*H*N minor), 7.38-7.28 (m, 5H *Ar* over total 9), 6.31-6.29 (m, 1H minor), 6.24-6.22 (m, 1H major), 6.12-6.07 (m, 2H major + minor), 4.30 (q, 1H, *J=6.7 Hz*), 2.82 (bb, 1H), 2.70-2.67 (m, 1H), 2.39 (ddd, 1H, *J=3.9 Hz*, *J=5.8 Hz*, *J=11.6 Hz*), 1.46 (dd, 3H, *J=5.6 Hz*, *J=6.7 Hz*), 1.45-1.41 (m, 2H), 1.33-1.27 (m, 2H), 1.01 (d, 3H, *J=7.9 Hz*), 0.84 (ddd, 1H, *J=2.7 Hz*, *J=9.4 Hz*, *J=11.6 Hz*) ppm.

^{13}C NMR [$CDCl_3$, 125 MHz, 20 °C]: major + minor products) δ 23.8 (CH_3), 25.0 (CH_3), 36.9 (CH_2), 43.4 (CH), 47.6 (CH_2), 47.8 (CH_2), 50.5 (CH), 53.9 (C_q), 69.2 (CH), 126.4 (CH*Ar*), 126.6 (CH*Ar*), 128.2 (CH*Ar*), 133.1 (HC=CH minor), 133.8 (HC=CH major), 138.7 (HC=CH major), 139.6 (HC=CH minor), 145.6 (C_{qAr}), 145.5 (C_{qAr}), 170.4 (C-imine major), 170.6 (C-imine minor).

IR [ATR]: the signal assigned as the C=O moiety of compound **3a** (peaks at 1720 cm^{-1}) decreased. A new peak assigned as the C=N moiety of compound **5b** appeared at 1660 cm^{-1}.

2-methyl-bicyclo[2.2.1]hept-5-en-2-ylmethylene-(1-cyclohexyl)-amine (5c)

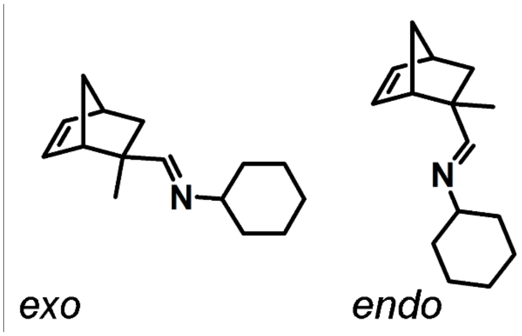

Conversion 96 % including the 22% of side product, obtained in significant amount from the reaction of **3a** with **4c**. Ratio *endo:exo* isomers of compound **5c** is of 26:74, calculated by ^{1}H NMR.

MS [ESI, positive mode]: peaks were observed with *m/z* 218.3 and 240.3 and were assigned as $C_{15}H_{24}N$ ($[M+H]^+$ predicted *m/z* 218.2) and $C_{15}H_{23}NNa$ ($[M+Na]^+$ predicted *m/z* 240.3), respectively.

[GC]: a peak was observed with *m/z* 217.2 at 16.0 min and was assigned as $C_{15}H_{24}N$ ($[M]^+$ predicted *m/z* 217.2).

^{1}H NMR [$CDCl_3$, 500 MHz, 20 °C]: (major + minor products) δ 7.73 (s, 1H, C*H*N major), 7.49 (s, 1H, C*H*N minor), 6.21 (dd, 1H, *J=5.6 Hz, J = 3.0 Hz*), 6.08 (dd, 1H, *J=5.6 Hz, J=3.1 Hz*), 2.96-2.91 (m, 1H), 2.81 (bb, 1H), 2.62 (bb, 1H), 2.29 (dd, 1H, *J=3.9 Hz, J=11.6 Hz*), 1.82-1.14 (m, 12H of the total 26), 0.96 (s, 3H), 0.81 (dd, 1H, *J=2.7 Hz, J=11.6 Hz*) ppm.

^{13}C NMR [$CDCl_3$, 125 MHz, 20 °C]: δ 23.9 (CH_3), 24.9 (CH_2), 25.6 (CH_2), 34.2 (CH_2), 34.3 (CH_2), 34.4 (CH_2), 37.1 (CH_2), 43.3 (CH), 47.8 (CH_2), 50.7 (CH), 69.8 (CH), 123.3 (C_q), 133.8 (HC=CH), 138.6 (HC=CH), 169.5 (C-imine), 169.6 (C-imine) ppm.

IR [ATR]: the signal assigned as the C=O moiety of compound **3a** (peaks at 1720 cm^{-1}) decreased. A new peak assigned as the C=N moiety of the side product of compound **5c** appeared at 1660 cm^{-1}.

(2-methyl-bicyclo[2.2.1]hept-5-en-2-ylmethyl)-phenyl-amine (6a)

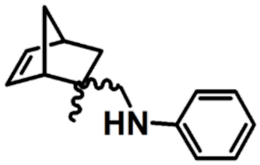

Reactivity 43 % of **3a** reacted.

R*f* 0.83 [SiO_2, EtOAc/Hexane = 2/8 (*v*/*v*)]

MS [ESI^+]: two peaks were observed with m/z 214.2, 255.2 and 277.1 and were assigned as $C_{15}H_{20}N^+$ ([M + H^+] predicted m/z 214.16), $C_{17}H_{23}N_2^+$ ([M + MeCN + H^+] predicted m/z 255.19), and $C_{17}H_{22}N_2$ Na^+ ([M + MeCN + Na^+] predicted m/z 277.17), respectively.
[GC]: peaks were observed with *m/z* 213.2 at 12.3 and 22.7 min and were assigned as $C_{15}H_{19}N$ ($[M]^+$ predicted *m/z* 213.1).

^{1}H NMR [$CDCl_3$, 400 MHz, 20 °C]: δ 0.90 (dd, 1H, *J=2.6 Hz, J=11.6 Hz*), 0.98 (s, 3H), 1.44 (ddd, 1H, *J=1.8 Hz, J=4.2 Hz, J=8.7 Hz*), 1.61 (dd, 1H, *J=3.7 Hz, J=11.6 Hz*), 1.65-1.68 (m, 1H), 2.60 (bb, 1H), 2.82 (bb, 1H), 3.16 (s, 2H), 3.67 (bb, NH), 6.11 (dd, 1H, *J=3.1 Hz, J=5.7 Hz*), 6.16 (dd, 1H, *J=2.9 Hz, J=5.7 Hz*), 7.64-7.61 (m, 2CH*Ar*), 6.66-6.70 (m, CH*Ar*), 7.15-7.20 (m, 2CH*Ar*) ppm.

^{13}C NMR [$CDCl_3$, 100 MHz, 20 °C]: δ 24.1 (CH_3), 38.8 (CH_2), 42.5 (C_q), 43.3 (CH), 48.1 (CH_2), 49.3 (CH), 55.1 (CH_2), 112.6 (CH*Ar*), 117.0 (CH*Ar*), 129.2 (CH*Ar*), 135.3 (HC=CH), 136.7 (HC=CH), 148.8 (C_q*Ar*) ppm.

IR [ATR]: the signal assigned as the C=O moiety of the aldehyde of the cycloadduct (peaks at 1720 cm^{-1}) decreased.

(2-methyl-bicyclo[2.2.1]hept-5-en-2-ylmethyl)-(1-phenyl-ethyl)-amine (6b)

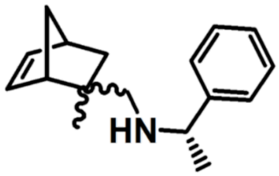

Reactivity 88 % of **3a** reacted.

R_f 0.83 [SiO_2, EtOAc/Hexane = 2/8 (*v*/*v*)] (detected using $KMnO_4$).

MS [ESI^+]: a peak was observed with m/z 264.4 and was assigned as $C_{17}H_{23}NaN^+$ ([M + Na^+] predicted m/z 264.17).

^{1}H NMR [$CDCl_3$, 500 MHz, 20 °C]: δ 7.35-7.29 (m, 4H *Ar*), 7.25-7.20 (m, 1H *Ar*), 6.10 (dd, 1H, *J=5.6 Hz, J=2.8 Hz*), 6.07 (dd, 1H, *J=5.6 Hz, J=2.7 Hz*), 3.72 (dq, 1H, *J=1.6 Hz, J=6.6 Hz*), 2.70 (bb, 1H), 2.60-2.50 (m, 2H), 2.41 (dd, 1H, *J=8.0 Hz, J=11.5 Hz*), 1.46-1.38 (m, 2H), 1.35 (dd, 3H, *J=1.9 Hz, J=6.6 Hz*), 1.33-1.26 (m, 2H), 0.90 (d, 3H, *J=4.1 Hz*), 0.77 (ddd, 1H, *J=2.5 Hz, J=9.4 Hz, J=11.5 Hz,*) ppm.

^{13}C NMR [$CDCl_3$, 125 MHz, 20 °C]: δ 24.0 (CH_3), 24.8 (CH_3), 39.1 (CH_2), 42.2 (C_q), 43.1 (CH), 47.8 (CH_2), 49.1 (CH), 59.0 (CH), 59.1 (CH_2), 126.6 (CH*Ar*), 126.7 (CH*Ar*), 128.3 (CH*Ar*), 135.5 (HC=CH), 136.5 (HC=CH), 146.4 (C_{qAr}) ppm.

IR [ATR]: the signal assigned as the C=O moiety of the aldehyde of the cycloadduct (peaks at 1720 cm^{-1}) decreased.

cyclohexyl-(2-methyl-bicyclo[2.2.1]hept-5-en-2-ylmethyl)-amine (6c)

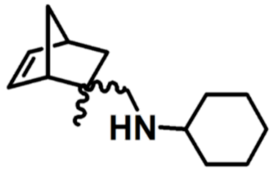

Reactivity 100 % of **3a** reacted.

R*f* 0.83 [SiO_2, EtOAc/Hexane = 2/8 (*v*/*v*)] (detected using $KMnO_4$)

^{1}H NMR [$CDCl_3$, 400 MHz, 20 °C]: δ 0.80 (dd, 1H, *J=2.6 Hz, J=11.6 Hz*), 0.89 (3H, s), 1.04-1.15 (m, 3H), 1.19-1.21 (m, 1H), 1.23-1.28 (m, 2H), 1.34-1.39 (m, 1H), 1.49 (dd, 1H *J=3.7 Hz, J=11.5 Hz*), 1.60-1.63 (m, 2H), 1.69-1.76 (m, 2H), 1.85-1.88 (m, 2H), 2.34 (tt, 1H *J=3.8 Hz, J=10.4 Hz*), 2.51 (bb, 1H), 2.64 (s, 2H), 2.75 (bb, 1H), 6.08 (dd, 1H *J=3.0 Hz, J=5.6 Hz*), 6.11 (dd, 1H *J=2.8 Hz, J=5.6 Hz*) ppm.

^{13}C NMR [$CDCl_3$, 100 MHz, 20 °C]: δ 14.1 (CH_3), 23.9 (C_q), 25.2 (CH_2), 26.3 (CH_2), 33.8 (CH_2), 39.1 (CH_2), 43.2 (CH), 47.9 (CH_2), 49.3 (CH), 57.9 (CH), 58.7 (CH_2), 136.1 (HC=CH), 136.5 (HC=CH) ppm.

MS [ESI^+]: a peak was observed with m/z 258.1 and was assigned as $C_{15}H_{25}KN^+$ ([M + K^+] predicted m/z 258.16)**.**
[GC]: peaks were observed with *m/z* 219.2 at 17.1 and 16.8 min and were assigned as $C_{15}H_{25}N$ ($[M]^+$ predicted *m/z* 219.2).

IR [ATR]: the signal assigned as the C=O moiety of the aldehyde of the cycloadduct (peaks at 1720 cm^{-1}) decreased.

General procedure for synthesis of the isolated tertiary amides

To a round bottom flask (25 mL) were added THF (5 mL), **6c** (1.0 mmol, 219 mg), triethylamine (1.0 mmol, 0.14 mL), and corresponding acid chloride (1.0 mmol). The reaction mixture was stirred for 20 min. and then quenched by addition of HCl (10 mL, 1.0 M, aq.) The product was extracted with ethyl acetate (30 mL), then organic phase was washed with saturated aqueous solution of $NaHCO_3$, and dried with Na_2SO_4 (anhydr.). The amide product was purified by column chromatography AcOEt/hexane (1/20) (*v/v*).

N-cyclohexyl-N-(((1S,4S)-2-methylbicyclo[2.2.1]hept-5-en-2-yl)methyl) propionamide

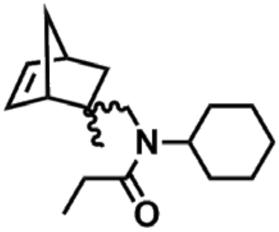

Yields 83 % (227 mg, 0.83 mmol).

R_f 0.10 [SiO_2, EtOAc/Hexane = 1/20 (*v*/*v*)]

^{1}H NMR [DMSO-*d*$_6$, 600 MHz, 70 °C]: δ (major + minor products) δ 6.22 (bb, 1H, minor), 6.20 (bb, 1H, minor), 6.15 (bb, 1H, major), 6.09 (bb, 1H, major), 3.38 (d, 1H, *J=15.3 Hz*), 3.34 (d, 1H, *J=15.3 Hz*), 2.75 (bb, 1H), 2.55 (bb, 1H), 2.32 (bb, 2H), 2.28 (bb, 2H), 1.81-1.49 (m, 10H), 1.36-1.18 (m, 3H), 1.17-1.05 (m, 1H), 1.05-0.96 (m, 3H major + 3H minor), 0.81 (bb, 3H minor), 0.78 (bb, 3H major) ppm.

^{13}C NMR [DMSO-*d*$_6$, 125 MHz, 20 °C]: δ 9.53 (CH_3), 23.3 (CH_3 major), 24.5 (CH_3 minor), 24.8 (CH_2), 25.6 (CH_2), 26.8 (CH_2), 40.2 (CH), 42.0 (CH), 42.2 (CH), 47.3 (CH_2), 47.4 (CH_2), 52.1 (CH_2), 53.1 (C_q), 57.7 (CH_2), 134.9 (HC=CH minor), 135.4 (HC=CH major), 136.6 (HC=CH major), 137.0 (HC=CH minor), 173.2 (C-amide), 173.7 (C-amide) ppm.

MS [ESI$^+$]: HRMS: $[C_{18}H_{29}NONa]^+$ calculated 298.2141, found 298.2146

***N*-cyclohexyl-*N*-((2-methylbicyclo[2.2.1]hept-5-en-2-yl)methyl)**

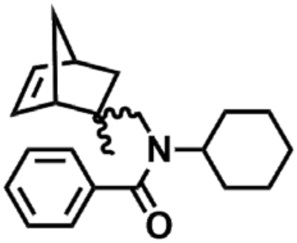

Yields 76 % (246 mg, 0.76 mmol).

R*f* 0.12 [SiO_2, EtOAc/Hexane = 1/20 (*v*/*v*)]

^{1}H NMR [DMSO-*d*6, 600 MHz, 80 °C]: δ (major + minor products) δ 7.46-7.41 (m, 3H major + 3H minor), 7.38-7.32 (m, 2H major), 7.32-7.29 (m, 2H, minor), 6.25 (bb, 1H minor), 6.19-6.14 (m, 1H minor), 6.15-6.12 (m, 1H, major), 6.10-6.05 (m, 1H major), 3.50 (d, 1H, *J=14.3 Hz*), 3.43 (d, 1H, *J=14.3 Hz*), 3.40 (bb, 1H major), 3.30 (bb, 1H, minor), 3.22 (d, 1H minor, *J=14.3 Hz*) 3.17 (bb, 1H major), 2.55 (bb, 1H), 3.16 (s, 1H major), 2.98 (d, 1H minor, *J=14.3 Hz*), 2.72 (bb, 1H major), 2.54 (bb, 1H major), 2.51-2.48 (m, 2H), 2.44 (bb, 1H minor), 1.85-1.45 (m, 10H), 0.85 (bb, 3H major + 3H minor), 0.81-0.75 (m, 1H) ppm.

^{13}C NMR [DMSO-*d*6, 125 MHz, 20 °C]: δ 23.8 (CH_3 major), 24.1 (CH_2 major), 24.5 (CH_2 major), 24.7 (CH_3 minor), 25.5 (CH_2), 30.7 (CH_2), 40.0 (CH), 40.7 (CH_2), 42.1 (C_q), 42.2 (CH), 42.4 (C_q), 47.3 (CH_2), 47.4 (CH_2) 51.2 (CH), 52.4 (CH), 59.4 (CH), 60.0 (CH_2), 125.9 (CH minor), 126.1 (CH major), 128.1 (CH minor), 128.2 (CH major), 128.6 (CH minor), 128.8 (CH major), 135.2 (HC=CH minor), 135.4 (HC=CH major), 136.7 (HC=CH major), 136.9 (HC=CH minor), 138.1 (C_q-Ar), 138.2 (C_q-Ar), 172.0 (C-amide), 172.9 (C-amide) ppm.

MS [ESI$^+$]: HR-MS: $[C_{22}H_{29}NONa]^+$ calculated 346.2147, found 346.2114

***N*-cyclohexyl-*N*-((2-methylbicyclo[2.2.1]hept-5-en-2-yl)methyl)-2-phenyl-acetamide**

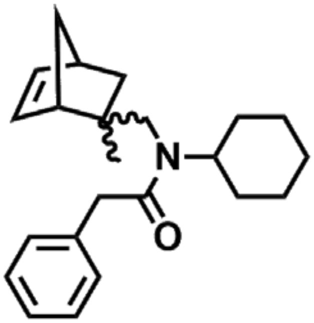

**Yields**	39 % (130 mg, 0.39 mmol).
$\mathbf{R_f}$	0.14 [SiO_2, EtOAc/Hexane = 1/20 (*v*/*v*)]
**^{1}H NMR**	[$CDCl_3$, 600 MHz, 20°C]: δ (major + minor products) 7.25-7.32 (m, 3H major + 3H minor), 7.20-7.24 (m, 2H major + 2H minor), 6.15-6.19 (m, 1H minor), 6.09-6.15 (m, 1H major + 1H minor), 6.04-6.09 (m, 1H major), 3.78 (bb, 2H major), 3.69 (s, 2H minor), 3.29-3.63 (m, 2H major + 2H minor), 2.83 (bb, 1H minor), 2.76 (bb, 1H major), 2.61 (bb, 1H major), 2.59 (bb, 1H minor), 2.22-2.35 (m, 2H major + 2H minor), 1.74-1.86 (m, 6H major + 2 minor), 1.52-1.67 (m, 6H major + 6H minor), 1.11-1.26 (m, 4H major + 4H minor), 0.99 (s, 3H minor), 0.93-0.98 (m, 2H minor), 0.87-0.92 (m, 2H minor), 0.77 (s, 3H major) ppm.
**^{13}C NMR**	[$CDCL_3$, 125 MHz, 20 °C]: δ (major + minor products) 23.7 (CH_3), 24.0 (CH_3), 25.4 (CH_2), 25.6 (CH_2), 26.3 (CH_2), 26.8 (CH_2), 29.3 (CH_2), 29.5 (CH_2), 32.4 (CH_2), 41.0 (CH_2), 42.5 (CH_2), 43.0 (CH_2), 43.3 (CH), 43.6 (CH), 47.8 (CH_2), 48.2 (CH_2), 51.0 (CH), 53.4 (CH), 57.2 (CH_2), 59.4 (CH_2), 61.6 (C_q), 63.0 (C_q), 126.6 (CH), 126.7 (CH), 128.7 (CH), 128.8 (CH), 128.9 (CH), 135.5 (CH), 135.8 (C_q), 135.9 (C_q), 136.1 (CH), 137.3 (CH), 172.1 (C_q), 172.5 (C_q) ppm.
**MS**	[ESI^+]: $[C_{23}H_{31}NONa]^+$ calculated 360.2303, found 360.2336

Supplementary Note 5

Mathematical discussion of the number of pathways

Our navigation approach uses decision making whereby only the most reactive pathway i.e. the most reactive reagent is chosen and the other possible pathways are rejected. By using this method, a combinatorial explosion of the number of possible reactions to be done is avoided. It also means that the fraction of reactions that need to be done, as a function of the number of reaction 'generations' and the number of reagents per generation, decreases dramatically as the number of generations or reagents per generation is increased. The number of reactions that need to be performed is given in **Supplementary Equation 6**. The total number of reactions to cover all possible reactions, (tn$R$) is given in **Supplementary Equation 7**. The number of reagents is (R) and the number of reaction generations is (G).

$$\mathrm{n}R = R\ x\ G \quad (6)$$

$$\mathrm{tn}R = \sum_{n=1}^{G} R^{n \to G} \quad (7)$$

As can be seen in **Supplementary Figure 5** there is a dramatic drop in the fraction of reactions that need to be done. As the number of generation and possible reagents is increased the fraction of reactions that would need to be done using our navigating algorithm decreases quickly.
